# Localized Cancer Treatment Using Thiol–Ene Hydrogels for Dual Drug Delivery

**DOI:** 10.1021/acs.biomac.5c00387

**Published:** 2025-04-08

**Authors:** Lakshmi Sathi Devi, Maria Rosa Gigliobianco, Serena Gabrielli, Dimitrios Agas, Maria Giovanna Sabbieti, Maria Beatrice Morelli, Consuelo Amantini, Cristina Casadidio, Piera Di Martino, Roberta Censi

**Affiliations:** † School of Pharmacy, ChIP Chemistry Interdisciplinary Project Research Centre, University of Camerino, Via Madonna delle Carceri, 62032 Camerino, MC, Italy; ‡ Department of Pharmacy, University of “G. D’Annunzio” Chieti and Pescara, Via dei Vestini 1, 66100 Chieti, CH, Italy; § School of Science and Technology, ChIP Chemistry Interdisciplinary Project Research Centre, University of Camerino, Via Madonna delle Carceri, 62032 Camerino, MC, Italy; ∥ School of Biosciences and Veterinary Medicine, University of Camerino, Via Gentile III da Varano, 62032 Camerino, MC, Italy; ⊥ School of Pharmacy, Department of Experimental Medicine and Public Health, University of Camerino, Via Madonna delle Carceri, 62032 Camerino, MC, Italy

## Abstract

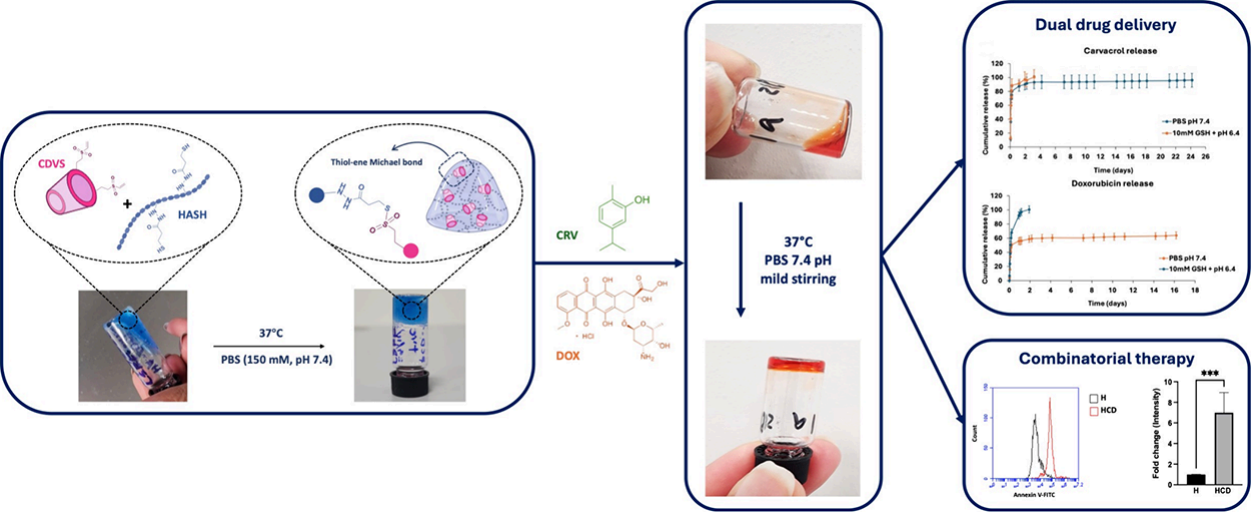

Combinatorial cancer therapy benefits from injectable hydrogels for localized, controlled drug delivery. This study presents a thiol–ene conjugated hydrogel formed by cross-linking thiol-modified hyaluronic acid (HASH) with vinyl sulfone-modified β-cyclodextrin (CDVS). Four formulations (23Gel-16, 23Gel-33, 99Gel-16, 99Gel-33) were synthesized by varying HASH molecular weight (23 or 99 kDa) and CDVS modification (16% or 33%). Rheological analysis confirmed enhanced viscoelasticity with increasing molecular weight and modification (99Gel-33 > 99Gel-16 > 23Gel-33 > 23Gel-16). The system enabled combinatorial delivery of doxorubicin (DOX) and carvacrol (CRV), exhibiting tumor-responsive degradation and tunable release. DOX release accelerated under tumor-mimicking conditions (100% in 46 h vs 58.7% in PBS), while CRV showed an initial burst followed by sustained release. The hydrogel promoted mesenchymal stem cell proliferation and effectively inhibited triple-negative breast cancer cells. This injectable, tumor-responsive hydrogel system offers a promising platform for minimally invasive, personalized cancer therapy.

## 1 Introduction

The well-known structural compatibility and biomimetic properties of hydrogels make them excellent carriers for biomedical applications, closely resembling the extracellular matrix and exhibiting mechanical properties similar to those of natural tissues.^1,2^ However, achieving optimal viscoelasticity and mechanical stability remains a key challenge, as these factors critically influence drug release kinetics and minimize mechanical irritation to tissues.^1,3^ The functional properties of hydrogels are shaped by variables such as polymer concentration, molecular weight, and cross-linking density, which collectively affect pore size, viscosity, swelling behavior, and mechanical strength.^4,5^ By tailoring these parameters, hydrogels with stable and tunable properties can be developed.^6^

Injectable *in situ* forming hydrogels represent a notable innovation in localized cancer therapy. Initially administered as a liquid, these hydrogels are directly injected into solid tumors, where they undergo a phase transition to form a gel depot, enabling the sustained release of anticancer agents.^7^ This technology allows localized drug delivery directly to the tumor site, providing a minimally invasive approach and thereby minimizing systemic exposure and side effects compared to systemic administration.^8^ Among cross-linking strategies, the Michael addition reaction stands out for its biocompatibility and efficiency, particularly in facilitating *in situ* hydrogel formation. The thiol–ene Michael addition reaction provides selective hydrogel formation under mild, biocompatible conditions, making it an ideal approach for engineering hydrogel-based delivery systems.^9,10^ Its chemo-selectivity ensures precise conjugation between thiols and alkenes without unwanted side reactions, minimizing the unintended cross-reactivity with biological structures. By offering definite control over the encapsulation efficiency and drug release kinetics, the thiol–ene conjugated hydrogels improve therapeutic efficacy and reduce adverse effects.^11−13^

Hydrogel systems based on modified hyaluronic acid (HA) and β-cyclodextrin (CD) are widely studied for cancer treatment due to the biocompatibility and hydration properties of HA, as well as the supramolecular chemistry of CD, which enables efficient drug encapsulation and controlled release.^14,15^ In a previous study, a modified HA/CD-based hydrogel, developed through a “click” reaction between thiol and vinyl sulfone groups, was explored for its antitumor efficacy by loading doxorubicin via host–guest interaction of CD. The CD-drug interaction resulted in a sustained release, and the system exhibited a dose-dependent cytotoxicity against MCF-7 cancer cells.^16^ However, from a therapeutic perspective, complex diseases such as cancer often involve multiple biological pathways, making their treatment challenging with single-drug therapies.^17,18^ Combinatorial therapy addresses this issue by simultaneously targeting these pathways, enhancing treatment quality, and reducing the risk of drug resistance. Even so, the effective co-administration of multiple drugs requires precise release synchronization to avoid dose mismatches or drug–drug interactions, which may arise with separate administrations.^19,20^ Taking this aspect into account, this study aims to develop *in situ* forming viscoelastic hydrogels by cross-linking thiol-functionalized hyaluronic acid (HASH) and vinyl sulfone-modified β-cyclodextrin (CDVS) via thiol–ene Michael addition. This research introduces a novel approach to evaluating the hydrogel's potential as a dual-drug delivery platform for both hydrophilic and hydrophobic therapeutics. Incorporating multiple drugs within a hydrogel system ensures controlled and synchronized release, reduces dosing frequency, and improves patient compliance.^21,22^ For instance, research by Li *et al*. demonstrated the potential of a sustained-releasing *in situ* hydrogel system, synthesized from four-arm polyethylene glycol thiol (PEGSH) and poly(ethylene glycol) diacrylate (PEGDA), for melanoma treatment, codelivering doxorubicin and imiquimod, which effectively suppressed tumor growth and metastatic progression by inducing immunogenic cell death and activating immune responses.^17^ Thus, combinatorial therapy stands as a superior alternative to multiple individual delivery methods.^23^

The novel hydrogel systems in this study were designed to exploit the chemo-selectivity and fast reaction rate of thiol groups toward vinyl sulfones to avoid unwanted cross-reaction with surrounding biocomponents involving hydroxyl or amine groups.^24−26^ The tailorable and viscoelastic properties of the developed hydrogels were thoroughly investigated in this research, by considering different hydrogel formulations composed by varying the molecular weight of HA and modification degree of CDVS, keeping the final polymer concentration constant. Comprehensive characterizations were performed to understand cross-linking efficiency, stability, swelling behavior, thermal properties, rheological tunability, mechanical performance, and cytotoxicity. The potential of combinatorial cancer therapy was evaluated for the first time in this system by loading hydrophilic doxorubicin hydrochloride (DOX) and hydrophobic carvacrol (CRV) as therapeutics. *In vitro* drug release studies in media mimicking physiological and tumor microenvironments, along with *in vitro* cell studies analyzing apoptosis and drug uptake by tumor cells, were conducted to investigate the antitumor activity of both released DOX and CRV.

## 2 Materials and Methods

### 2.1 Materials

All the chemicals were purchased and used as received from Sigma-Aldrich (Schnelldorf, Germany), unless otherwise stated. Sodium hyaluronic acid (HA) of molecular weights 23 kDa and 99 kDa was purchased from Contipro (Dolní Dobrouč, Czech Republic) and Altergon Italia Srl (Avellino, Italy), respectively. β-cyclodextrin (CD) was supplied by A.C.E.F. S.p.A. (Piacenza, Italy). The 3,3′-dithiobis(propanoic dihydrazide) (DTP) was previously synthesized using the method described by Vercruysse *et al*.^27^ Culture dishes and 48-well culture plates were purchased from Euroclone (Milano, Italy). Minimum essential medium (MEM), fetal bovine serum (FBS), penicillin, and streptomycin were provided by Gibco (ThermoFisher, Milano, Italy). CellTiter 96 AQueous One Solution Cell Proliferation Assay (MTS) kit was purchased from Promega (Milano, Italy). Distilled water was obtained with the apparatus RO 60 TS demi2 water deionizer provided by Gamma 3 Ecologia (Castelverde Costa Sant’abramo, Italy). The phosphate-buffered saline (PBS), with NaCl (136.9 mM), KH_2_PO_4_ (1.7 mM), and Na_2_HPO_4_ (13.4 mM), was used as a pH 7.4 physiological buffer. The PBS with NaCl (140.31 mM), NaH_2_PO_4_ (20.84 mM), and Na_2_HPO_4_ (17.61 mM) was used as a pH 6.4 acidic buffer.

### 2.2 Polymers Synthesis

#### 2.2.1 Synthesis of Thiolated Hyaluronic Acid (HASH)

The HA polysaccharide was modified with thiol groups following the synthesis protocol described in previous papers with slight modifications.^28,29^ The functionalization was performed on HA of molecular weights 23 and 99 kDa separately, denoting them as 23-HASH and 99-HASH, respectively.

First, 500 mg of HA was dissolved in distilled water (50 mL for 23 kDa and 100 mL for 99 kDa, respectively) along with DTP (0.85 and 0.63 mmol for 23 kDa and 99 kDa, respectively) at room temperature, followed by pH adjustment to 4.75 using 1 M HCl. The 1-ethyl-3-(3-(dimethylamino)propyl) carbodiimide hydrochloride (EDC.HCl) (molar ratio of DTP to EDC equals 1:1) was then added as a carbodiimide agent, which will activate the carboxyl groups of HA to form an intermediate o-acylisourea ester, which will react with the amino groups in DTP. The reaction was kept under stirring at room temperature, maintaining 4.75 pH for 48 h. The pH was then increased to 7 using 1 M NaOH to stop the reaction, and dithiothreitol (DTT) (21.74 and 5.05 mmol for 23 kDa and 99 kDa, respectively) was added to reduce the disulfide bonds. The solution was further stirred for 24 h, keeping a stable pH of 8.5 at room temperature. After that, the pH of the final solution was reduced to 3.5 and dialyzed (Molecular weight cut off = 12–14 kDa) against NaCl solution (100 mM, pH 3.5) for 2 days at 4 °C, and then against distilled water for another 2 days at 4 °C.

The final yield for both batches was obtained as a white spongy product by lyophilization and stored at −20 °C. The percentage of thiol groups per carboxyl units of each HA mol, defined as the degree of substitution (DS%), was calculated from ^1^H NMR in D_2_O and revealed peaks at δ 3.3–4.6 (protons of the HA backbone), 2.01 (3H of NHCOC**H**_**3**_), 2.7 (2H of C**H**_**2**_CH_2_SH), and 2.85 (2H of CH_2_C**H**_**2**_SH).

#### 2.2.2 Synthesis of Vinyl Sulfonated Β-Cyclodextrin (CDVS)

For the vinyl sulfone modification of CD, the synthesis procedure was followed with slight modifications, as described in previous papers.^30,31^ In this work, CDVS with DS% of approximately 16 and 33% was synthesized, denoted as CDVS_16 and CDVS_33, respectively. To exemplify the typical reaction, the synthesis of CDVS_33 is reported.

Two grams of vacuum-dried CD were dissolved in 32 mL anhydrous dimethylformamide (DMF) under stirring. The reaction system was set in an oil bath at 50 °C. Subsequently, 24 mL triethylamine (TEA) was added, followed by a slow dropwise addition of 3.5 mL divinyl sulfone (DVS), and the reaction mixture was left to stir at 50 °C for 24 h. Dried sodium-activated Dowex ion-exchange resin was then added to remove the unwanted triethylammonium ions. After 1 h of continuous stirring at room temperature, the resins were vacuum-filtered. The desired product was then precipitated in 500 mL of precooled diethyl ether, filtered under vacuum, washed with acetone (3 × 30 mL), and then vacuum-dried at room temperature. The final product was characterized by ^1^H NMR in D_2_O. The DS% was calculated based on the peak integrals of the CD proton (1H) at δ 5.1 to the vinyl protons at δ 6.3–6.5 (2H, C**H**_**2**_=CH–SO_2_) from ^1^H NMR. Other signals include δ 3.3–4.4 (4H, C**H** of pyranosyl and 2H, −CH–C**H**_**2**_–OH of glucose) and δ 6.8 (1H, CH_2_=C**H**–SO_2_).

#### 2.2.3 Proton Nuclear Magnetic Resonance Spectroscopy (^1^H NMR)

The confirmation of the chemical structure and the determination of the degree of substitution (DS%) of the synthesized polymers, HASH and CDVS, were done using the 500 MHz ^1^H NMR spectrometer (Ascend 500, Bruker, Massachusetts, United States) using deuterium oxide (D_2_O) as a solvent. Chemical shifts were referred to the solvent peak (δ = 4.79 ppm for D_2_O), and the obtained spectrum was evaluated using MestReNova software (version 14.2.1).

### 2.3 Placebo Hydrogel Preparation

Hydrogels with a final volume of 100 μL and a total polymer concentration of 6% w/v were formulated by separately dissolving HASH and CDVS in 150 mM PBS pH 7.4 at room temperature. Once dissolved, the CDVS solution was added dropwise to the HASH solution under continuous mild stirring to obtain a 1:1 molar ratio between thiol and vinyl sulfone groups, and then the mixture was kept stationary in the incubator at 37 °C. The gelation was monitored every 10 min until the polymer mixture solution came to an immovable state by the inverted tube technique, generating a no-flow system upon tilting.^32,33^ Four different hydrogel combinations (23Gel-16, 23Gel-33, 99Gel-16, and 99Gel-33) were prepared as denoted in Table 1, where the combinations differ in their HA polymer molecular weight (23,000 Da vs 99,000 Da) and the degree of substitution (16% vs 33%) of the CDVS. As controls, two HASH hydrogels (23Gel and 99Gel) were prepared in the absence of CDVS, according to the concentrations described in Table 1, using only disulfide cross-linking chemistry.

**Table 1:** Overview of the Composition of the Hydrogel Formulations under Study

**hydrogels composition**		**polymers**	***M***_**W**_**(Da)**^a^	**DS%**	**conc.** (% w/v)	**final conc.** (% w/v)
**Michael addition cross-linked hydrogels**	**23Gel-16**	23-HASH	23,000	45	2.8	6.0
		CDVS_16	1135	16	3.2	
	**23Gel-33**	23-HASH	23,000	45	3.9	6.0
		CDVS_33	1135	33	2.1	
	**99Gel-16**	99-HASH	99,000	45	2.7	6.0
		CDVS_16	1135	16	3.3	
	**99Gel-33**	99-HASH	99,000	45	3.8	6.0
		CDVS_33	1135	33	2.2	
**disulfide cross-linked hydrogels (controls)**	**23Gel**	23-HASH	23,000	45	3.9	3.9
	**99Gel**	99-HASH	99,000	45	3.8	3.8

a *M*_W_ = molecular weight, provided by the suppliers.

#### 2.3.1 Raman Spectroscopy

Raman spectroscopy was done on the lyophilized hydrogel and synthesized polymers to clarify the existing chemical cross-linking. Raman scattering was conducted using a micro-Raman spectrometer (iH320, Horiba, Japan) in backscattering geometry. Raman imaging of HASH was done by setting an argon-ion laser of 532 nm emission wavelength and that of CDVS and the lyophilized hydrogel was done using an infrared IR laser of 785 nm emission wavelength as the excitation light source and an Olympus microscope (mod. BXFM-ILHS, Olympus Corporation, Tokyo, Japan) equipped with a 50X long working distance objective. The resultant scattered light was dispersed using a diffraction grating number of 600 grooves per millimeter onto a thermoelectric-cooled CCD detector (Horiba Syncerity, Horiba, Japan).

#### 2.3.2 Thermal Analysis (TGA and DSC)

The hydrogel behavior and composition were studied under the influence of temperature using differential scanning calorimetry (DSC). Analyses were performed to investigate the complexation efficacy of the employed method using a DSC 250TA Instruments (New Castle, USA) equipped with a cooler RCS90, using about 2 mg of the dried sample placed into a concave aluminum pan with a pierced lid under the nitrogen gas flow of 50 mL/min. All samples were subjected to the thermal program of first heating from −10 to 350 °C, which was performed at a rate of 10 °C/min. The instrument was calibrated with an indium standard. On the other hand, thermogravimetric analysis (TGA) was carried out using a TGA 55 TA (New Castle, USA), previously calibrated with a nickel standard. Briefly, 10 mg of sample was heated from room temperature to 650 °C, under a nitrogen atmosphere of 50 mL/min with a heating rate of 10 °C/min for both dried lyophilized and wet hydrogel samples.

#### 2.3.3 Scanning Electron Microscopy (SEM)

The morphology and pore size of the dehydrated hydrogels were studied using a field emission scanning electron microscope (FE-SEM Zeiss Sigma 300, Zeiss, Germany). The samples were placed in a double-sided adhesive carbon tape in an aluminum stub and coated with an approximately 10 Å thickness chromium layer using a sputtering system (Quorum Q150T ES, Quorum Technologies, Lewes, UK). Prior to the analysis, the hydrogels were allowed to swell for 24 h and were dried overnight using lyophilization.

#### 2.3.4 Hydrogel Mechanical Properties

The flow behavior and the mechanical properties of the hydrogels can be studied under external force application, as they directly depend on the physical properties like cross-linking density and molecular weights. Rheological characterization was done on the formulated hydrogels using a modular compact rheometer, MCR 92 (Anton Paar GmbH, Graz, Austria). The instrument controlling and input-output data analysis were managed by Anton Paar RheoCompass software. Throughout the experiments, the temperature was preset and kept constant at 37 °C unless mentioned otherwise. Rheological analyses were carried out using a two-plate model in a parallel plate system with a diameter of 50.0 mm, and the gap was set to 0.1 mm. Experiments were performed on Michael addition cross-linked hydrogels (Table 1) with a final volume of 500 μL.

A shear-rate-controlled test was conducted at a temperature of 25 °C using ascending logarithmic steps to study the viscosity behavior. Viscosity was measured as a function of the applied shear rate of 0.1 to 100 1/s. The hydrogels were subjected to the frequency sweep test to study the change in their viscoelastic behavior with varying angular frequencies from 0.1 to 100 rad/s at a constant oscillating shear strain of 1%, isothermally. In addition, more oscillatory model tests were done to analyze the stress–strain property at a constant angular frequency of 10 rad/s and an oscillating shear strain range from 0.01 to 100%. The mean elasticity value within the linear viscoelastic region was plotted for all the samples.

Furthermore, a time-dependent deformation at constant stress was studied by performing a creep-recovery test, isothermally, following a two-step stress method. A constant shear stress of 50 Pa was applied to the hydrogels for exactly 300 s (5 min), and then, the hydrogels were allowed to relax for 600 s (10 min) at zero-shear stress, and the released strain was recorded.^34^

#### 2.3.5 Hydrogels Swelling Behavior

For swelling studies, 100 μL of hydrogel samples prepared as described in section 2.3 (Table 1) were weighed initially, then supplemented with 900 μL of 150 mM phosphate-buffered saline (PBS) at pH 7.4 and incubated at 37 °C. At predetermined time intervals, all the PBS was carefully removed from the gel surface. The weight of the hydrogels was recorded, followed by the addition of fresh PBS. The experiments were run in triplicate.

The swelling ratio was calculated using eq 1:
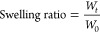
1where the final weight of the swollen hydrogels at time *t* after each time interval is denoted as *W*_*t*_ and the initial weight of the hydrogels is denoted as *W*_0_.

#### 2.3.6 Hydrogels Redox-Stability Studies

The stability of the chemical cross-linking of the hydrogels was evaluated by a control test under a reductive environment. In this case, we conducted the test on 23Gel-33 and 99Gel-33 hydrogels with their respective controls, 23Gel and 99Gel, as mentioned in section 2.3 (Table 1). For better visual quality, the 150 mM PBS (pH 7.4) was colored using dextran blue dye while 0.435 M of DTT was used as the reducing agent.^35^ The concentration of DTT used was the highest concentration required to ensure the cleavage of the disulfide bonds during the synthesis of HASH, as described in section 2.2.1.

Hydrogels composed of HASH and CDVS were prepared in the same way as described previously in section 2.3, at the final volume of 100 μL and concentration of 6% w/v. At the same time, 100 μL of 23Gel and 99Gel were formulated in PBS at a final concentration of 3.9% w/v and 3.8% w/v, respectively, and incubated at 37 °C for 1 h. Such hydrogels composed of only HASH prepared without the CDVS moiety are the controls. Further, all the hydrogels were supplemented with 900 μL of 0.435 M DTT solution and monitored at regular time intervals of 5 min. At each interval, the DTT solution was removed from the hydrogel surface, and the hydrogels were weighed before being supplemented with fresh 0.435 M DTT solution. The experiments were run in duplicates. The swelling ratio was calculated according to eq 1, as mentioned in section 2.3.5.

#### 2.3.7 *In Vitro* Hydrogel Degradation Studies

The degradation behavior of the 99Gel-33 hydrogel was tested in four different media labeled: PBS pH 7.4, enzyme, 10 mM GSH+ pH 6.4 + enzyme, and 10 mM GSH + pH 6.4. While PBS pH 7.4 mimics the physiological environment, the latter three media mimic different conditions of the tumor microenvironment. The enzyme media was prepared by dissolving hyaluronidases (150 U/mL) in acidic buffer pH 6.4; the 10 mM GSH + pH 6.4 + enzyme media by dissolving 150 U/mL hyaluronidases and 10 mM GSH in acidic buffer pH 6.4; and the 10 mM GSH by dissolving 10 mM GSH in acidic buffer pH 6.4. Placebo 99Gel-33 hydrogels of 100 μL volume were prepared as explained in section 2.3, in triplicates for each medium. After noting the initial weight of the gels, 900 μL of the freshly prepared media was added and incubated at 37 °C. At scheduled time intervals, the supernatants were removed carefully, and the weight of each gel was taken and supplemented with new supernatants. The degradation was calculated as recovered weight using eq 1 described above (section 2.3.5).

#### 2.3.8 *In Vitro* Hydrogel Cytocompatibility

Bone marrow-derived mouse mesenchymal stem cells (MSCs) were obtained as previously described^36,37^ and grown in 100 mm culture dishes in a minimum essential medium (α-MEM; Life Technologies, Monza, Italy) supplemented with 10% heat-inactivated fetal calf serum (HIFCS) (Life Technologies, Monza, Italy), penicillin, and streptomycin (Sigma-Aldrich, Milano, Italy) at standard conditions (37 °C, 5% CO_2_). After reaching 80% of confluence, the MSCs cells were plated on 48-well culture dishes previously coated with 6% w/v of 99Gel-33 or 23Gel-33 (following section 2.3) and incubated for 5 days, refreshing the medium after 3 days. Three types of controls were used: (1) plating the cells in hydrogel-free wells; (2) cell-free wells coated with 6% w/v of 99Gel-33; and (3) cell-free wells coated with 6% w/v of 23Gel-33, following the same timing as described above. After 5 days of incubation, the metabolic activity of viable cells was determined by the MTS [3-(4,5-dimethylthiazol-2-yl)-5-(3-carboxymethoxyphenyl)-2-(4-sulfophenyl)-2H-tetrazolium] assay following the manufacturer's instructions.^38^ The colored formazan product resultant was measured by reading the absorbance at 490 nm TECAN reader (Tecan Italia s.r.l., Cernusco Sul Naviglio (MI), Italy).

### 2.4 Drug-Loaded Hydrogel Formulation

#### 2.4.1 CDVS-CRV Complexation and Gas Chromatography

The encapsulation of hydrophobic CRV inside the CDVS cavity was performed using the previously described method.^39,40^ Briefly, 36.32 mg CDVS_33 was dissolved in 2 mL distilled water, and CRV (molar ratio of CD to CRV is 1) was dissolved in 2 mL ethanol separately. The CRV-ethanol solution is then added dropwise into the CDVS solution. The obtained mixture was kept under stirring in the dark overnight, while covered with open holes to allow ethanol evaporation. Further, the final dried product was collected by lyophilization with a final yield of 98% based on the initial weight of CDVS. Quantification was performed using GC to evaluate the encapsulation efficiency (EE) and the loading capacity (LC) of the CRV-loaded CDVS complexes.

To obtain high-sensitivity quantification of CRV, an Agilent 8890 gas chromatograph (GC) coupled with an Agilent 5977/B mass selective detector (MSD) was employed with an Agilent 7650 autosampler (Santa Carla, California, USA). The system was equipped with a separation column of Agilent HP-5 (30 m × 250 μm × 0.25 μm) at a constant flow of 1.2 mL/min. Helium was used as the carrier gas in splitless injection mode at an injector temperature of 250 °C and septum purge flow at 3 mL/min. The oven was set with an initial temperature of 40 °C with a 2 min hold, then raised to 160 °C (0 min hold) at a rate of 20 °C/min, and finally, the temperature was increased to 300 °C (0.3 min hold) at 100 °C/min, with a total run time of 9.7 min. The mass spectrometer was operating in electron impact mode with 70 eV ionization energy. The MSD transfer line, ion source, and quadrupole temperatures were 250, 230, and 150 °C. Selected ion monitoring (SIM) mode was used to perform the analysis using the ions *m*/*z* 91.10, 135.10, and 150.00. For reference, calibration was performed on known concentrations of CRV ranging from 0.01 to 50 ppm.

#### 2.4.2 DOX/CRV-Loaded Hydrogel Formulation

To prepare the DOX/CRV-loaded 99Gel-33 hydrogel, 99-HASH (3.8% w/v) was dissolved in the PBS solution of 200 μg DOX, and CDVS-CRV complex (from section 2.4.1) (2.2% w/v) was dissolved in PBS separately, followed by the dropwise addition of the latter into the former solution under gentle stirring. The loaded gelling solutions resulted in a final volume of 100 μL and a concentration of 6% w/v. The final mixture was incubated at 37 °C, and gelation was assessed over a period of 1 h until the solution flow became immobile using the inverted tube method.^41^

#### 2.4.3 *In Vitro* Release Experiment

To evaluate the combinatorial delivery efficiency of the 99Gel-33 hydrogels loaded with dual drugs (DOX and CRV), an *in vitro* release experiment was set up in the media labeled PBS (pH 7.4) and 10 mM GSH + pH 6.4 mimicking the physiological and tumor microenvironment, respectively. Both the media were prepared in the presence of 10% (v/v) ethanol to favor the solubility of lipophilic CRV. The DOX/CRV-loaded hydrogels of 100 μL were supplemented with 900 μL of the media and placed at 37 °C in an incubator. At prescheduled intervals, aliquots were withdrawn and replaced with fresh media. The collected aliquots were used for further analysis of drug concentration. DOX was quantified using a fluorescence microplate reader (FLUOstar Omega, BMG LABTECH, Ortenberg, Germany) at excitation/emission wavelengths of 480/590 nm by placing 200 μL of the aliquots in a 96-well plate. GC was employed for CRV quantification by dissolving 100 μL of the withdrawn aliquots in 1 mL acetone (section 2.4.1). The study was performed in triplicates.

#### 2.4.4 Rheological Analysis of DOX-Hydrogel Interactions

The mechanical properties of the blank and DOX-loaded 99Gel-33 hydrogels were analyzed in duplicates using a modular compact rheometer, MCR 92 (Anton Paar GmbH, Graz, Austria), to investigate the possible interactions between the hydrogel and DOX. The storage (*G*′) modulus representing the elasticity of both hydrogels was investigated by conducting a frequency test at an oscillating shear strain of 1% in the angular frequency ranging from 0.1 to 100 rad/s. Experiments were performed isothermally at 37 °C on hydrogels of volume 500 μL using a parallel plate system with a diameter of 50 mm.

### 2.5 *In Vitro* Drug Antitumor Activity

The experiments were carried out on the established MDA-MB-231 triple-negative breast cancer cell line (ATCC, LGC Standards, Milan, Italy). Primary human stabilized NHF A12 fibroblast cell line (ATCC, LGC Standards, Milan, Italy) was used as nontumoral cells. MDA-MB 231 and NHF A12 were cultured in Dulbecco’s Modified Eagle’s Medium (DMEM) supplemented with heat-inactivated fetal bovine serum (FBS, 10% v/v), penicillin (100 IU/mL) and streptomycin (100 μg/mL), and maintained at 37 °C with 5% CO_2_ and 95% humidity.

#### 2.5.1 Cell Viability Assay

The MTT [3-(4,5-dimethylthiazolyl-2)-2, 5-diphenyltetrazolium bromide] assay was used to assess the effect of drug solutions and drug-loaded 99Gel-33 hydrogels on MDA-MB 231 and NHF A12 cell lines (the latter used as a control healthy cell line). The cells were plated in 96-well plates (3 × 10^3^ cells/well) and, after 24 h, exposed to DOX/CRV-loaded 99Gel-33 hydrogels conditioned media containing the released drugs at prescheduled time points (4, 24, 48, and 72 h). To prove that the drugs released preserved their biological activity, fresh media containing equivalent free drug concentration (as described in the results section 3.3.1) were used as controls. After 24 and 48 h of incubation, 0.8 mg/mL of MTT was added to each well and further incubated for 3 h at 37 °C. Then, the culture medium was removed, and 100 μL DMSO/well was added to dissolve the formazan crystals. A microplate reader (Molecular Devices, Spectra Max ID3, München, Germany) measured the absorbance at 540 nm. Data were processed in GraphPad Prism 9.0.1(128) software (GraphPad Software, San Diego, CA, USA).

#### 2.5.2 Confocal Laser Scanning Microscopy (CLSM)

MDA-MB 231 cells were plated in a 96-well culture plate (3 × 10^3^ cells per well) at 37 °C overnight. After incubation, the medium was replaced by DOX/CRV-loaded 99Gel-33 hydrogels conditioned medium, and cells were incubated for up to 2 h at 37 °C to assess selective cellular internalization. To compare the cellular uptake in DOX/CRV-loaded 99Gel-33 hydrogels conditioned medium, a free DOX-/CRV mixture (having the same concentration of the drugs released) was added into the media and incubated for the same time. The cells were then washed with PBS and fixed with 4% paraformaldehyde. Cell nuclei were stained with PureBlu Hoechst 33342 Nuclear Staining Dye (BioRad, Milan, Italy). C2 Plus confocal laser scanning microscope (Nikon Instruments, Firenze, Italy) and NIS Element Imaging Software (Nikon Instruments) were used for the acquisition and processing of data.

#### 2.5.3 Annexin V Staining Assay

Flow cytometric analysis was performed to evaluate the cell death. MDA-MB 231 cells (2.5 × 10^4^ cells/mL) were seeded in 24-well plates. After overnight incubation, the medium was replaced by DOX/CRV-loaded 99Gel-33 hydrogels conditioned medium or free DOX/CRV mixture (having the same concentration of the drugs released), and cells were incubated at 37 °C for 2 h. Afterward, the treated cells were incubated with 5 μL/well Annexin V-FITC for 10 min at room temperature and analyzed by a BD Accuri C6 Plus flow cytometer (BD Biosciences, Milan, Italy) and its software.

### 2.6 Statistical Analysis

GraphPad Prism 9.0.1(128) software (GraphPad Software, San Diego, CA, USA) was used for statistical analysis. The results represent the mean ± standard deviation (SD) of three experiments. The statistical significance was determined using the Student’s test with two-tailed distribution for cell viability assay and the Welch’s *t* test for cytofluorimetric assay, where *p < 0.05 values were considered statistically significant (*p < 0.05, **p < 0.01, ***p < 0.001, ****p < 0.0001).

## 3 Results and Discussion

### 3.1 Polymer Synthesis and Chemical Characterization

**Figure 1:**
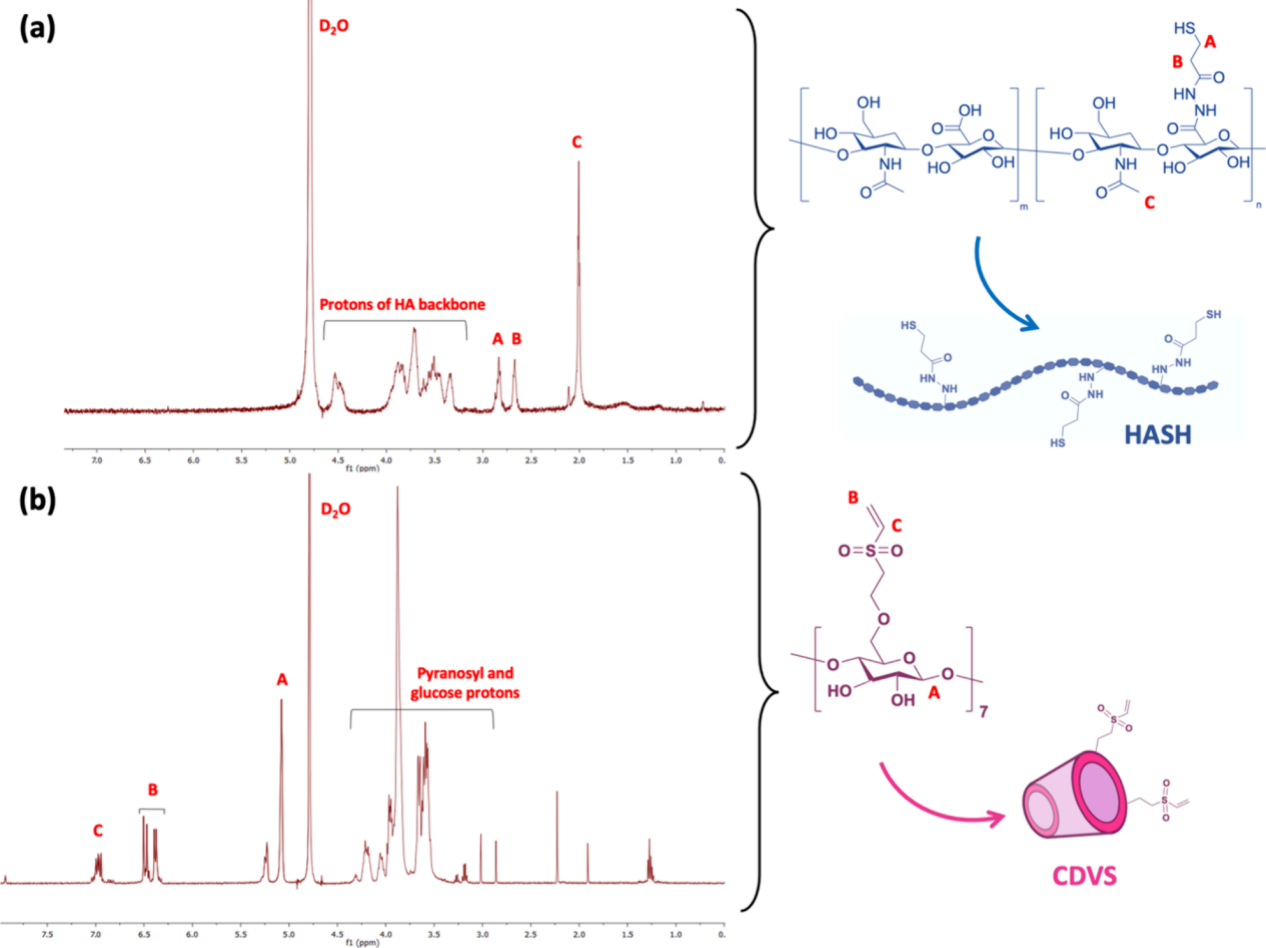
Representative ^1^H NMR spectra of (a) HASH and (b) CDVS in D_2_O. Polymer derivatizations were calculated following the methods described in sections 2.2.1 and 2.2.2.

This study details the successful synthesis of thiol-functionalized hyaluronic acid (HASH) and vinyl sulfone-modified cyclodextrin (CDVS) polymers. The thiol derivatization chemistry involves the linkage of HA with DTP via carbodiimide-mediated amide formation, followed by the reduction of disulfide bonds using DTT, giving free terminal thiol groups on each polymer unit of HA chains. We obtained a reproducible yield of around 95% for both 23-HASH and 99-HASH. From the ^1^H NMR (Figure 1a), the functionalization of the product was confirmed, and the DS%, defined as the percentage of the free carboxyl groups modified with thiols, was calculated around 45% for both 23-HASH and 99-HASH.

The modification of CD with vinyl sulfone (VS) moieties was done through its reaction with DVS in DMF as an organic solvent under the influence of TEA as a catalyst. These VS moieties could then react with the nucleophilic thiol groups of HA to introduce Michael-addition cross-linking. The first attempts were made by Fiorica *et al.*(31) by introducing divinyl sulfones on CD with a DS of about 33% and a final yield of 80%, followed by their conjugation with an amino derivative of HA to produce a cell/drug-releasing hydrogel system. Thus, along with the innate encapsulation purpose of CD via inclusion complexation, the modification also made it function as a cross-linker. In this study, VS functionalization was successfully done with a final product yield of 90%. The two resulting batches of CDVS, namely CDVS_16 and CDVS_33, exhibited DS% values of approximately 16 and 33%, respectively, as confirmed by ^1^H NMR measurements, with DS% defined as the percentage of free hydroxyl groups modified with vinyl sulfone moieties (Figure 1b).

The modification of HA and CD to introduce thiol and vinyl sulfone groups, respectively, provides the chemical foundation for Michael-addition-based cross-linking. The selective reactivity of thiol groups with vinyl sulfone ensures minimal cross-reactivity with biological components, enhancing biocompatibility and drug stability.^12^ In the context of Michael addition reactions, thiols, amines, and hydroxyl groups can all act as nucleophiles and react with electron-deficient alkenes (enes). However, thiols are highly reactive with enes due to the larger size and greater polarizability of the sulfur atom compared to the oxygen in hydroxy groups, allowing for better stabilization of the negative charge in the transition state, resulting in faster reaction rates. Primary and tertiary amines can also catalyze thiol–ene reactions effectively, though they require several hours to achieve high conversion, with their reactivity influenced by basicity and steric hindrance around the nitrogen atom. In contrast, hydroxy groups are less reactive in Michael addition reactions due to the oxygen atom’s lower polarizability, necessitating more stringent conditions such as higher temperatures or strong bases for effective reactions.^25,42^ Thus, although the hydroxyl or amine groups can work well in the Michael addition reactions, their low reactivity could lead to a slow cross-linking rate causing premature polymer dissolution for in situ hydrogel applications and possible cross-reactivity with the abundant amine groups present on proteins, drugs, and/or biological tissues, proving the suitability of thiols as the chosen nucleophile. Su *et al.* reviewed thiol-mediated chemo-selective strategies for the in situ formation of hydrogels, highlighting the potential for selectivity in these systems.^12^

### 3.2 Placebo Hydrogels: Formulation and Chemical Characterizations

**Figure 2:**
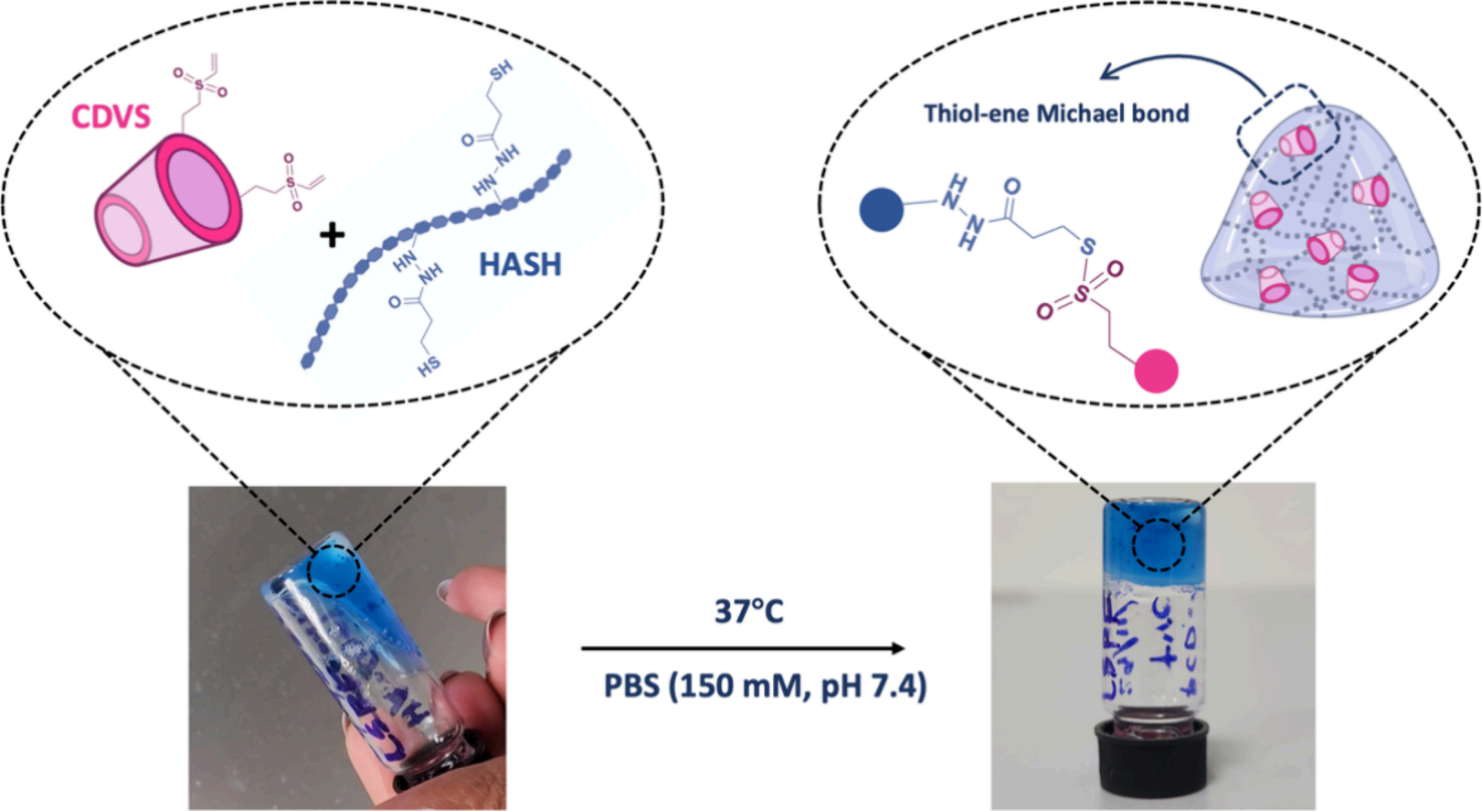
Schematic representation of the mechanism behind HA/CD-based hydrogel formation following Micheal addition reaction at pH 7.4 and at 37 °C. For better visual quality, the 150 mM PBS (pH 7.4) was colored using dextran blue dye.

Reacting the thiol and vinyl sulfone moieties under mild conditions led to the formation of thiol–ene Michael addition cross-linking, resulting in hydrogel production (Figure 2). The thiol–ene mechanism is a highly efficient and specific reaction used to form covalent bonds between a thiol (R–SH) and an electron-deficient alkene, like vinyl sulfones.^43^ The mechanism begins with the activation of the thiol, leading to the formation of a thiolate anion (R–S^–^). This thiolate anion then acts as a nucleophile, attacking the β-carbon of the vinyl sulfone groups, forming a carbon–sulfur bond. This nucleophilic addition process produces a stable Michael adduct, effectively incorporating the thiol across the double bond of the alkene.^11^ Vinyl sulfone groups are highly efficient Michael acceptors due to the sulfone’s electron-withdrawing nature, making them good electrophiles.^24^ Besides the chemo-selectivity and high reactivity, this reaction chemistry becomes practically interesting due to its biocompatible nature and the requirement of mild conditions involving just a physiological state of 37 °C and pH 7.4 with no use of external toxic catalysts.

Four batches of hydrogels were prepared as mentioned in section 2.3 (see Table 1). From preliminary insights, we observed that the rate of gelification is directly proportional to the molecular weights of HA used. Hydrogels prepared with the higher molecular weight HA demonstrated the fastest gelation times: 99Gel-33 achieved gelation in just 1 h, and 99Gel-16 gelled in 8 h. In comparison, the lower molecular weight HA required more time to form gels, with 23Gel-33 completing gelation in 12 h and 23Gel-16 taking approximately 2 days to fully gel. Additionally, throughout gelification, both 23-HASH and 99-HASH formed gels more rapidly when reacted with the higher modified CD (CDVS_33) than with the lower modified CD (CDVS_16), consistent with the observed gelation times. Spectroscopic techniques involving FTIR and Raman spectroscopy were employed to understand the chemistry behind gel formation. For this, 99Gel-33 was chosen, representing all the hydrogels, to perform the spectroscopic analysis, since all the hydrogels were developed following the same preparation methods. The chemical composition of the polymers and hydrogels was studied by comparing the FTIR spectra of CDVS_33, 99-HASH, and lyophilized 99Gel-33 (Figure S1) as described in the Supporting Information. Although the FTIR outcome reveals the successful presence of both CD and HA in the hydrogel composition, the more relevant peaks, such as those of thiols, vinyl sulfones, and Michael addition bond required to comment on the cross-links, were not observed, possibly because of their poor absorption intensity.^44,45^

**Figure 3:**
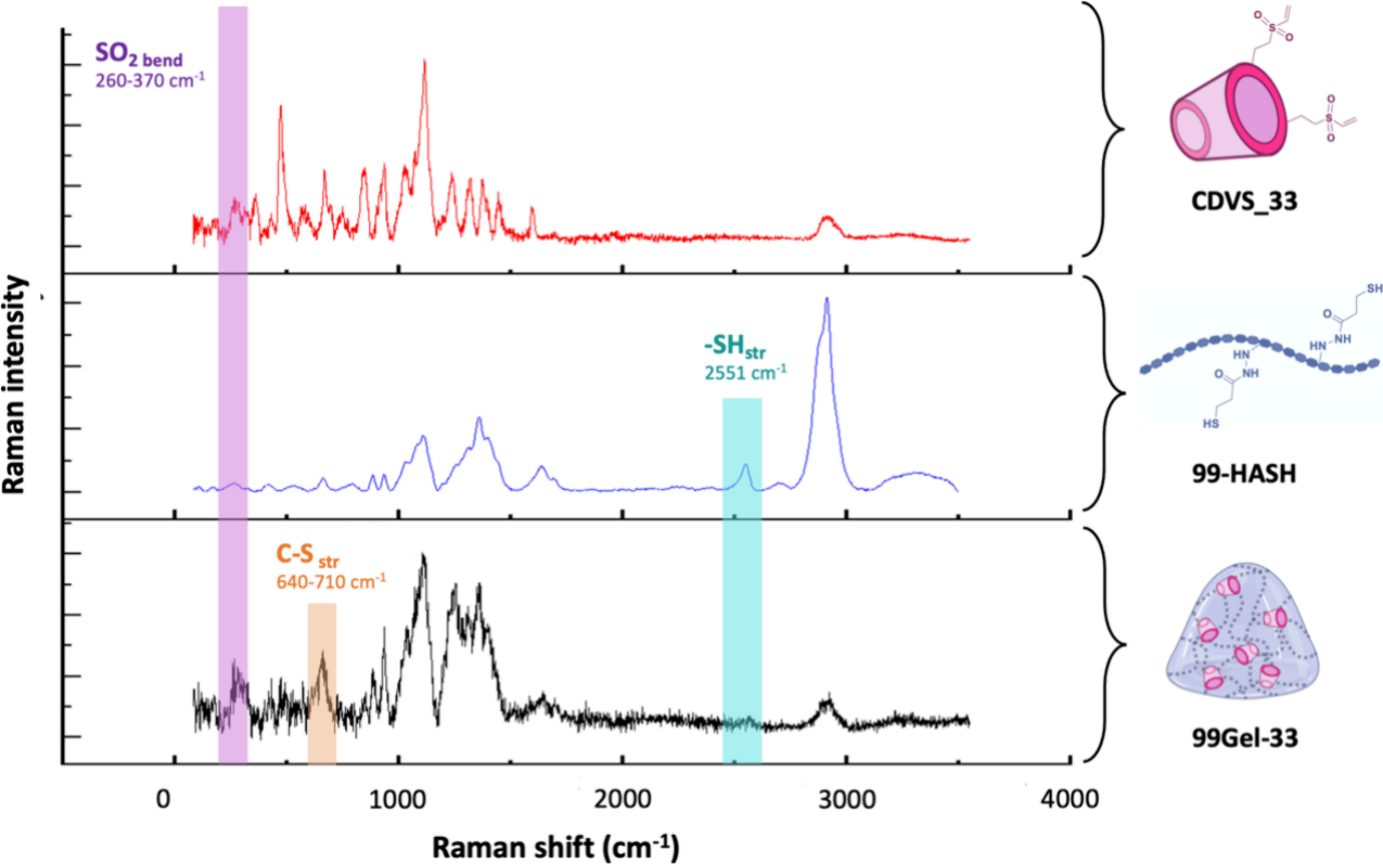
Raman spectra of CDVS_33, 99-HASH, and 99Gel-33, between 50 and 3500 cm^–1^. The measurements were performed on dried samples.

More detailed clarification and confirmation regarding the thiol–ene Michael addition occurrence were obtained from Raman spectroscopy as revealed in Figure 3. The Raman spectrum of 99-HASH revealed the presence of thiol groups at 2551 cm^–1^ (attributed to the stretching vibration of the −S–H bond) along with the characteristic signals of HA.^46^ When comparing the 99Gel-33 spectrum with that of 99-HASH, the former showed most of the characteristic signals of HA, confirming its dominant presence in the gel, similar to the FTIR observation. In addition, the disappearance of the thiol peak at 2551 cm^–1^ can also be seen from the 99Gel-33 spectrum. Furthermore, the presence of signals around 260–370 cm^–1^ in the hydrogel can be associated with the bending vibration of the −SO_2_ bond of the vinyl sulfone groups by comparison with CDVS signals.^47^ Additionally, the appearance of the signal at around 640–710 cm^–1^ in the gel is assigned to the new −C–S– Michael bonds between the thiol and vinyl sulfone groups.^44^ Briefly, the Michael addition cross-linking behind the gelification is proved by the disappearance of thiol groups, bending vibration of sulfone groups, and the appearance of −C–S– Michael bonds on gel formation.^47^

The efficiency of the thiol–ene reactions in the in situ formation of hydrogels is attractive as they allow the delivery of polymer precursors in combination with cells and soluble drugs in aqueous solutions through injection, resulting in the formation of 3D functional hydrogel networks at desired locations.^10,12^ In a study by Fu and Kao, in situ forming poly(ethylene glycol)-based hydrogels were successfully formulated via thiol-maleimide Michael addition, where they demonstrated the rapid reaction kinetics between maleimide and thiol functional groups in hydrogel formation, indicating efficient polymerization. The study further concluded that the incorporation of cells and sensitive compounds in hydrogels can be improved without the need for UV or other energy sources commonly used in biomedical hydrogel formation.^48^ Thus, the Michael addition-based gels offer a noninvasive administration of long-acting depots, avoiding the need for surgeries, hospitalizations, or specialized equipment.

#### 3.2.1 Polymers and Hydrogels Thermal Analysis

The differential scanning calorimetry (DSC) and thermogravimetric analysis (TGA) measurements were performed to study the response of the polymers and cross-links present in the hydrogels (23Gel-16, 23Gel-33, 99Gel-16, and 99Gel-33 formulations, composition Table 1) under the influence of temperature.

**Figure 4:**
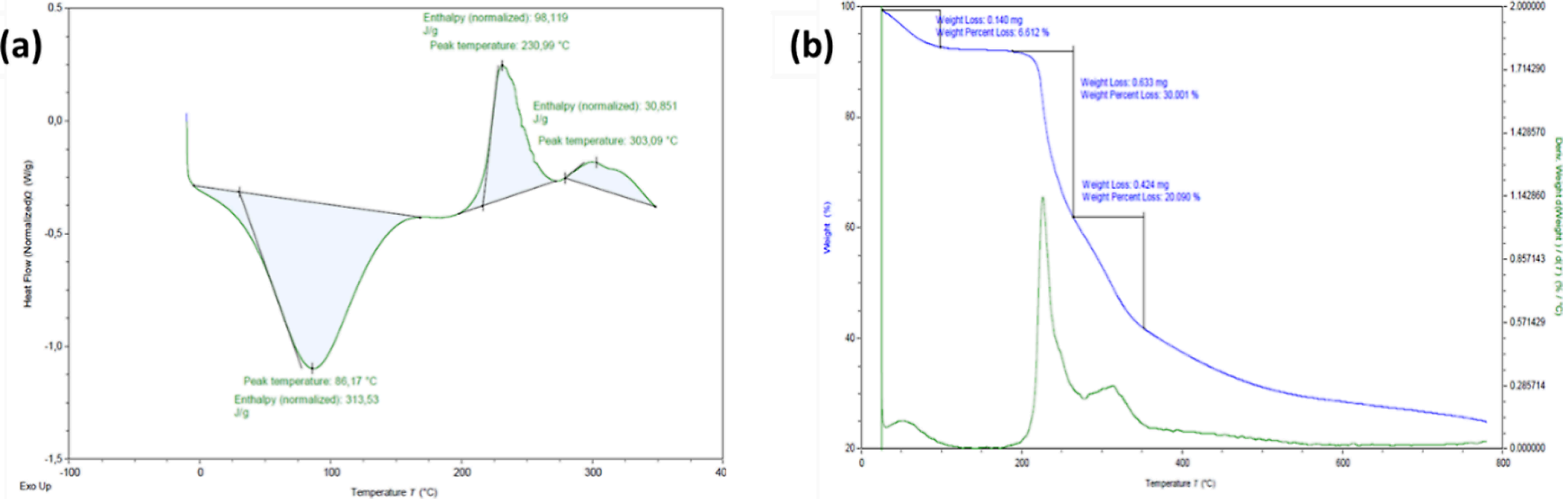
Thermal analyses results of lyophilized 99Gel-33 from (a) DSC and (b) TGA.

DSC was done on the lyophilized hydrogel samples. The typical observations from all the hydrogel samples are represented by a representative graph, shown as the DSC curve of 99Gel-33 in Figure 4a. The DSC analyses of the 23Gel-16, 23Gel-33, and 99Gel-16 formulations can be found in the Supporting Information and the individual precursor polymers CDVS_33 and 99HASH (Figures S3–S7, Supporting Information). The hydrogel sample showed an endothermic peak between 25 and 110 °C in the first heating, which is attributable to the dehydration process. Furthermore, two exothermic peaks are seen at around 230 °C and above 300 °C that can be relative to the degradation of HA and CD, respectively.^49,50^ It is assumed that the Michael addition bond present in the hydrogels breaks before reaching a temperature of 200 °C. This phenomenon explains the observation of two distinct exothermic degradation peaks corresponding to HA and CD. Elevated temperatures have been shown to induce thiol–ene retro-Michael reactions, as demonstrated by Zhang *et al.*, in which case the thiol-phenylvinylketone adducts showed retro-Michael activity at a temperature of at least 90 °C.^51^

TGA analysis was carried out on both lyophilized and hydrated hydrogel samples. Figure 4b shows the TGA curve of 99Gel-33 to represent the typical behavior of all the samples (23Gel-16, 23Gel-33, and 99Gel-16 formulations can be found in Figures S8–S10, Supporting Information). Table 2 summarizes the weight loss data of the lyophilized samples. All the samples show three weight losses: the first caused due to the volatile fraction of the bound water content, and the second and third due to the degradation of HA and CD, respectively.^52^ The degradation behavior and the onset temperatures are in correspondence with the DSC observations. A higher water loss percentage means lower water retention ability of the hydrogel. By comparing the hydrogels of CDVS_16 with those of CDVS_33, the amount of water loss was found to be more in the gels of CDVS_16 than in the gels of CDVS_33. In other words, higher levels of modification led to increased cross-linking density, resulting in a lower percentage of water loss. Table 3 summarizes the total water loss data of the hydrated samples. It is observed that 23Gel-33 showed the lowest percentage of water loss compared to the other samples. This is because 23Gel-33, with the highly modified short polymer chain of low molecular weight HA, offers a closely packed network structure. A study by Enev *et al*.^53^ explained that higher dry contents determined by TGA for hydrogels prepared from low molecular weight HA compared to high molecular weight HA might indicate more effective cross-linking of the low molecular weight HA networks, leading to less interstitial space. This smaller internetwork space limits the amount of water loss and tends to retain the water within the hydrogel structure. Thus, the TGA observation from Table 3 reveals the restricted interstitial space of hydrogel 23Gel-33 to escape the water content bound within the hydrogel matrix.

**Table 2:** TGA Weight Loss Data of the Lyophilized Hydrogels

	**first step weight loss**		**second step weight loss**		**third step weight loss**	
**hydrogel**	water (%)	onset^a^ (°C)	HASH (%)	onset^a^ (°C)	CDVS (%)	onset^a^ (°C)
**23Gel-16**	9.60	26.57	14.70	216.29	36.57	285.57
**23Gel-33**	7.17	25.36	28.87	218.78	18.43	303.41
**99Gel-16**	10.36	24.21	19.21	218.57	29.87	295.78
**99Gel-33**	6.61	40.80	30.00	219.02	20.09	255.69

a Onset = the temperature at which the initial degradation begins.

**Table 3:** Total Water Loss Data of the Hydrated Hydrogels

**hydrogel**	**first step weight loss of water (%)**
**23Gel-16**	90.75
**23Gel-33**	80.77
**99Gel-16**	90.66
**99Gel-33**	89.75

#### 3.2.2 Scanning Electron Microscopy (SEM)

**Figure 5:**
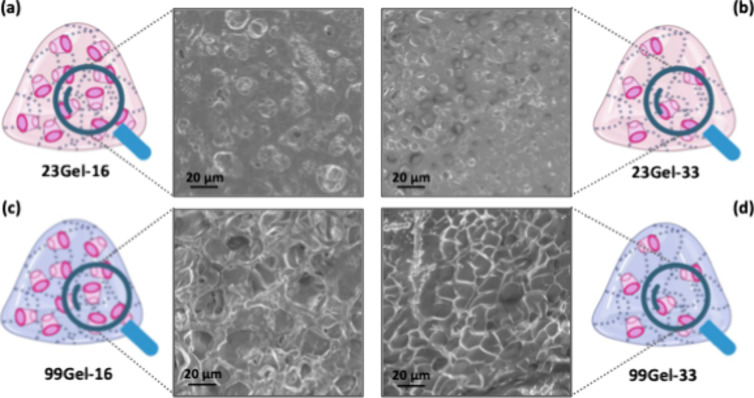
SEM images of (a) 23Gel-16, (b) 23Gel-33, (c) 99Gel-16, and (d) 99Gel-33.

The scanning electron microscopy (SEM) images of the four dehydrated 23Gel-16, 23Gel-33, 99Gel-16, and 99Gel-33 formulations (composition described in Table 1), revealed a highly porous morphology with varying mesh sizes depending on the degree of modifications of CDVS and the HA molecular weight (Figure 5). The SEM image of dehydrated 23Gel-33, as seen in Figure 5b, showed smaller-sized pores compared to the other samples. Furthermore, comparing hydrogels with the same molecular weight but varying degrees of modification revealed that those with higher modification levels exhibit reduced mesh sizes compared to their less modified counterparts. Indeed, dehydrated 23Gel-16 exhibits larger pores compared to dehydrated 23Gel-33 (Figure 5a,b), and a similar trend is observed for dehydrated 99Gel-16, which shows larger pores compared to dehydrated 99Gel-33 (Figure 5c,d). This reduction in mesh size correlates with a decreased capacity for water absorption in the highly modified hydrogels compared to those with lower modification rates. These data support the results obtained from the stability test and thermal analysis. Since SEM analysis requires hydrogels to be dehydrated, the dehydration process could affect their structure and morphology. To overcome this limitation and better understand their structural and mechanical properties, rheological analysis was performed in the hydrated state (section 3.2.3). This approach provides valuable insights into the hydrogel behavior under physiological conditions, ensuring a more thorough characterization of their properties in both dry and hydrated forms.

#### 3.2.3 Hydrogel Mechanical Characterization

Optimizing the design, formulation, and efficiency of drug delivery systems is a critical aspect of pharmaceutical development. Rheological characterization serves as a valuable tool in this process, providing insights into the flow and deformation properties of materials that directly influence the performance and reliability of these systems.^54,55^ In this study, rheological analysis of the synthesized hydrogels (23Gel-16, 23Gel-33, 99Gel-16, 99Gel-33, compositions described in Table 1) was conducted to elucidate their physicochemical properties and assess their potential for tunability.

**Figure 6:**
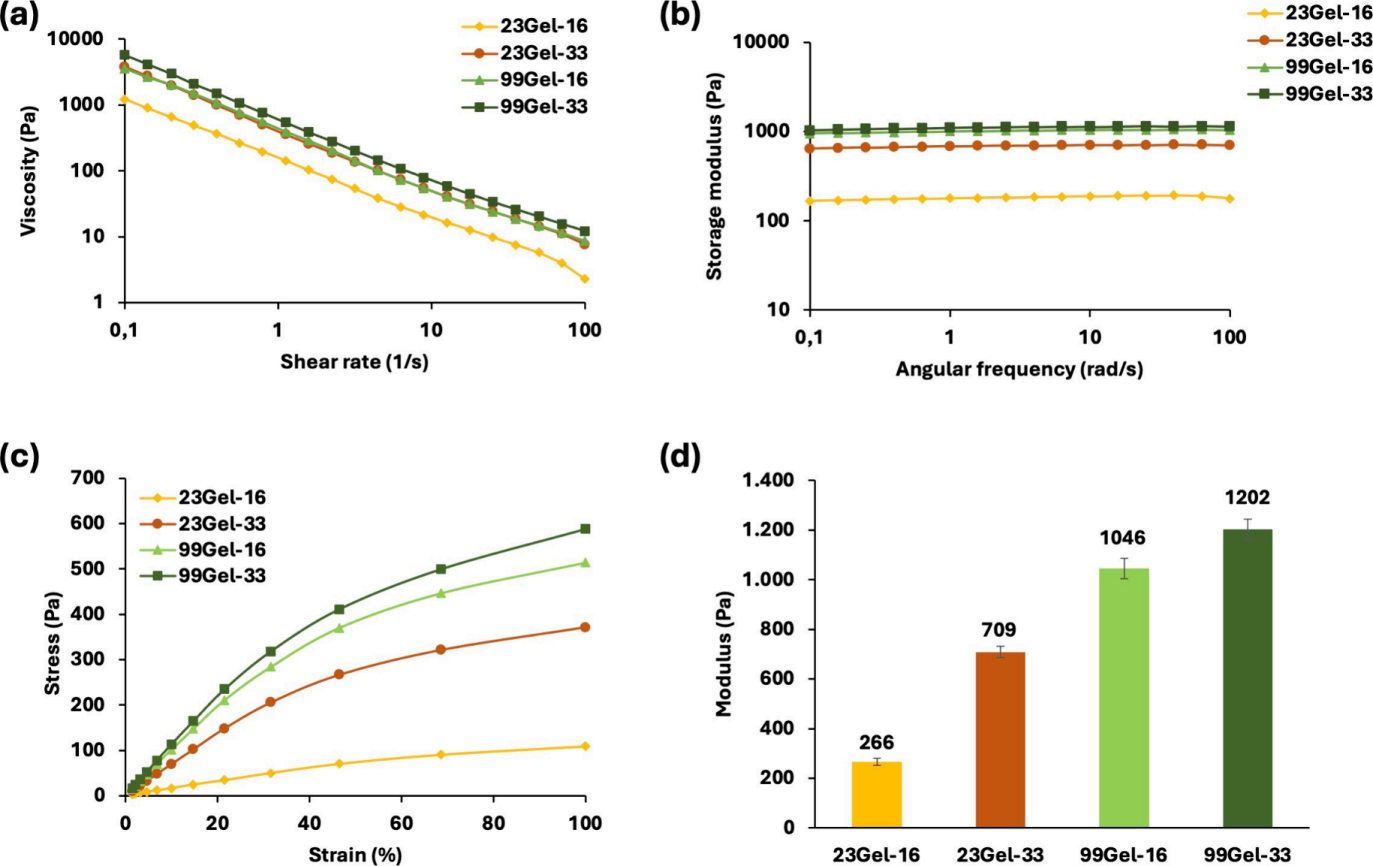
Graphical outcome of the hydrogels from the rheological studies of (a) viscosity vs shear rate at room temperature (25 °C); (b) storage modulus vs angular frequency at 37 °C; (c) stress–strain behavior at 37 °C; and (d) elastic modulus within the LVE range at 37 °C.

As shown in Figure 6a, the viscosity of the hydrogels was found to decrease with increasing shear rate, displaying a distinct shear-thinning flow behavior. However, the plateau phase, characterized by a constant viscosity as the shear rate approaches zero, was not observed. This is likely because the plateau region occurs at shear rates below 0.1 s^–1^ for our hydrogels.^56^ To address this, we extrapolated the data to establish a relationship between the flow behavior and the molecular mass distribution of the samples. This was achieved by fitting the experimental data to the Carreau model of viscosity, as described in eq 2(57):
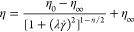
2where η, γ̇, η_0_, λ, *n*, and η_∞_ represent viscosity, shear rate, zero shear viscosity, relaxation time, shear thinning index, and infinite shear viscosity, respectively. The values were determined using Rheology Lite software (version 1.1.3.0). For shear-thinning materials, the *n* value, which is the measure of how strongly the material exhibits shear-thinning behavior, should be less than one (*n* < 1).^57^ As shown in Table 4, all the hydrogels exhibited *n* values less than one. Additionally, η_0_ is the viscosity at zero shear rate where the polymer chains are completely entangled and at rest, which means it has a proportional relation to the molecular mass distribution.^58,59^ In brief, materials with higher molar mass distribution will have higher zero shear viscosity due to their high friction and low mobility with a longer relaxation time, causing a shorter shear thinning rate. Table 4 shows the trend in the values of η_0_, λ, and *n* with increasing molecular weight and modification rate. More specifically, the values of η_0_ and λ are the lowest for 23Gel-16 and the highest for 99Gel-33. Conversely, 23Gel-16 exhibits the highest *n* value, indicating greater shear-thinning behavior, while 99Gel-33 displays the lowest *n* value, reflecting a more Newtonian-like flow behavior. Thus, the lower the molecular mass distribution, the lower the relaxation time, the faster the shear thins, and the higher the viscous behavior would be. This reveals the high elastic response and stable structure of 99Gel-33.

**Table 4:** Summarized Values of Carreau Model Parameters Exhibited by Different Hydrogels under Study

	**Carreau parameters**		
**hydrogel**	**zero shear viscosity η_0_ (kPa s)**	**thinning index *n***	**relaxation time λ (s)**
**23Gel-16**	90.0	0.116	1321.4
**23Gel-33**	350.1	0.104	1810.0
**99Gel-16**	382.3	0.101	1865.3
**99Gel-33**	893.5	0.094	3025.7

Frequency tests are generally conducted to study the mechanical properties of hydrogels, where the material is subjected to oscillatory strain at varying frequencies while measuring the resulting stress.^60^ To comment on the viscoelastic nature of a material, the value of storage or elastic modulus (*G*′) is highly relevant as it directly correlates to the viscoelastic properties. A highly cross-linked structure can be considered more elastic and mechanically rigid.^34,54,60^ For all the hydrogel samples, *G*′ was higher than the loss or viscous modulus (*G*″), indicating the dominant elastic response over the viscous (Figure 6b). This type of material, where *G*′ > *G*″, is said to be gel-like or viscoelastic solids. Figure 6b shows the measured values of *G*′ of the hydrogel samples by varying angular frequency. A trend was observed where 99Gel-33 shows higher *G*′ values while 23Gel-16 shows the lowest. This trend further exhibits how the molecular weight and degree of modification can directly influence the cross-link density and the elasticity of the hydrogels.

The strain sweep test was conducted to further investigate the viscoelastic behavior of the hydrogels by applying increasing oscillatory strain at a constant frequency. This test generated a stress–strain graph, providing insights into how the hydrogel network responds to compression. The results revealed that, for a given strain percentage, hydrogels with a higher density of cross-links required greater stress, indicating their enhanced resistance to deformation due to their more robust network structure.^61^ For example, from Figure 6c, for a given strain of 68.5%, a stress of 90 and 499 Pa was observed for 23Gel-16 and 99Gel-33, respectively. With increasing cross-link density comes a more coordinated structure. So, a higher force is required to exert damage on the highly cross-linked hydrogel. The linear viscoelastic region (LVE region), which is the range where the test can be done without breaking the sample structure, was determined primarily to measure the elastic response within this range.^62^Figure 6d shows the elastic modulus of the hydrogels within their LVE region, and it follows around the same trended values obtained from the frequency test, where the highest *G*′ value was shown by 99Gel-33. When 99Gel-33 showed a modulus around 1202 Pa, 23Gel-16 showed around 266 Pa. Within the LVE range, the polymer entanglement within the hydrogel structure forces it to return to its initial conformation once the stress is removed.^34^ For a highly coordinated material, it will be rather difficult to show more strain due to its strong core. Thus, it is distinctly clear how elasticity highly depends on and varies by tuning the molar mass distribution and the cross-linking degree.

Furthermore, the mesh size (ξ) and cross-linking density (*n*_e_) of the hydrogels were determined based on rheological measurements (following the eqs S1 and S2 described in the Supporting Information). The results indicate that increasing the storage modulus (*G*′) leads to a decrease in mesh size and an increase in cross-linking density. Specifically, for 23Gel-16, the cross-linking density was found to be 0.07 mol/m^3^, with a mesh size of ∼28.9 nm. This suggests that the hydrogel has a relatively loose network structure. In comparison, 23Gel-33 showed an increased cross-linking density of 0.28 mol/m^3^, and a smaller mesh size of ∼18.2 nm, indicating a denser network. For the 99Gel series, 99Gel-16 exhibited a cross-linking density of 0.41 mol/m^3^ and a mesh size of ∼16.0 nm, reflecting a further increase in the density of the polymer network. The highest values were seen in 99Gel-33, with a cross-linking density of 0.44 mol/m^3^ and the smallest mesh size of ∼15.5 nm, suggesting the tightest and most compact polymer structure among all the samples. These results demonstrate that increasing the HA molecular weight and degree of functionality of CD-VS leads to higher cross-linking density and smaller mesh size, indicating more tightly connected polymer networks, as also confirmed by the swelling studies described in section 3.2.4.

**Figure 7:**
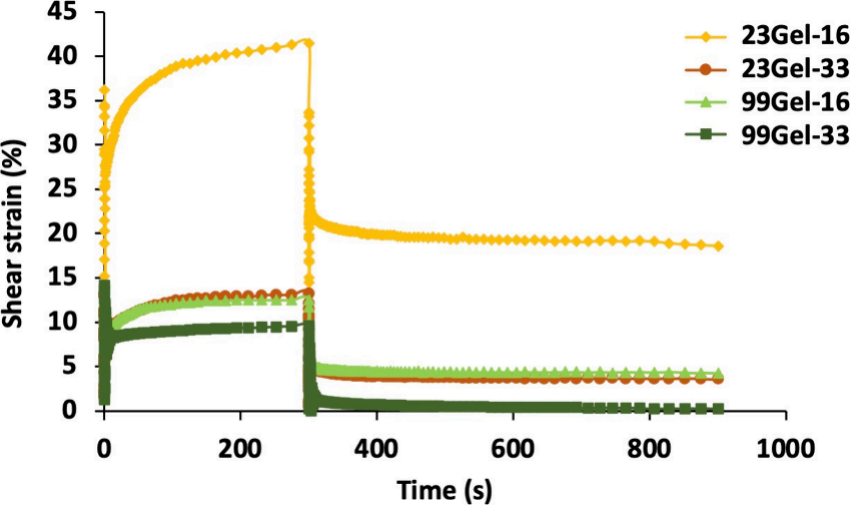
Creep and recovery behavior of the different hydrogels.

Creep test is an important rheological analysis, which measures the degree of deformation or strain experienced by the material under the application of constant stress over time. Understanding the creep behavior of hydrogels is crucial as it provides insights into the hydrogel behavior under long-term stress, which is vital for predicting the lifespan of the drug delivery system.^60,63^ The viscoelasticity, durability, and performance of a polymeric biomaterial can be directly commented on by the creep test. The test was done on different hydrogels by applying a constant stress of 50 Pa for 5 min, and their viscoelastic strain recovery was measured by releasing the applied stress for another 10 min. The strain recovery was measured by using the eq 3(34):
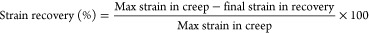
3Under applied stress, after 5 min, the hydrogels 23Gel-16 and 99Gel-33 exhibited strains of 41.5 and 9.6%, respectively (see Figure 7). When a constant force is applied to the hydrogel surface, localized strain develops, which can deform the gel network. This strain is primarily governed by the structural rigidity of the material.^64^ Highly cross-linked hydrogels, such as 99Gel-33, exhibit minimal strain, highlighting their high coordination and structural integrity. Conversely, 23Gel-16, with its weaker core and lower cross-linking density, demonstrates greater molecular mobility within its polymer chains. As a result, even minor stress induces significant deformation, amplifying the viscous behavior over the elastic properties of the hydrogel.

The viscoelastic recovery of the hydrogels was evaluated after the removal of applied stress. As shown in Table 5, 99Gel-33, which exhibited lower strain, demonstrated a higher recovery rate of 97%, as anticipated. In contrast, 23Gel-16 displayed the lowest recovery rate of 55%, highlighting its reduced ability to regain its original structure postdeformation. The study concludes that the hydrogel with higher coordination and strong intermolecular networks exhibits a lower strain on the application of force and has a higher tendency to take back its original structural conformation.

**Table 5:** Summarized Data of Creep-Recovery Test of the Hydrogels

**hydrogel**	**strain (%) under stress after 5 min**	**recovery (%) on releasing stress after 10 min**
**23Gel-16**	41.5	55
**23Gel-33**	13.2	69
**99Gel-16**	12.5	65
**99Gel-33**	9.6	97

#### 3.2.4 Hydrogel Swelling Studies

**Figure 8:**
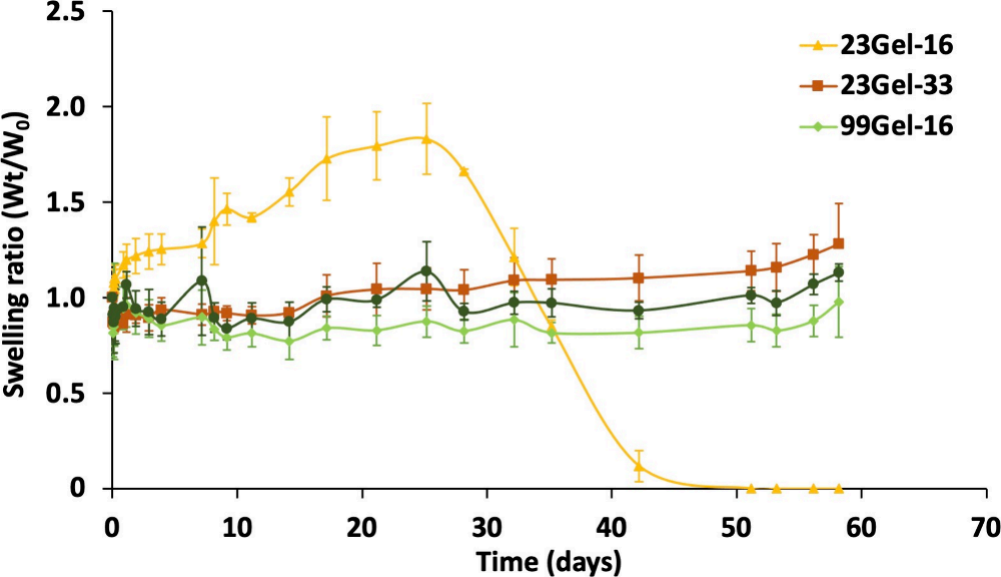
Swelling behavior of the 23Gel-16, 23Gel-33, 99Gel-16, and 99Gel-33 formulations in physiological environment at 37 °C (pH 7.4, 150 mM). All values are expressed as mean ± SD (*n* = 3).

The swelling behavior of the formulated hydrogels was studied by the addition of PBS (150 mM, pH 7.4) on top of them at 37 °C, followed by their weight change monitoring. The swelling ratio was calculated from the weight of the swollen hydrogel at each time interval (*W*_*t*_) and compared to its initial weight (*W*_0_). From Figure 8, it was observed that all the hydrogels except 23Gel-16 showed similar behavior against PBS and remained stable over time. While 23Gel-33 consists of shorter HA chains with a higher cross-linking density, resulting in a more compact structure, both 99Gel-16 and 99Gel-33 are composed of longer, heavier HA chains with extensive modifications and cross-linking. On the other hand, a sudden increase in swelling followed by weight loss was seen in 23Gel-16. Indeed, the composition of 23Gel-16, featuring low molecular weight HA and a lower cross-linking density, creates a more spacious network capable of absorbing and retaining a larger volume of water. This structural arrangement results in a high swelling ratio, with values approaching 2. This internal stress compromised the cross-linking and hydrogel network integrity of 23Gel-16, ultimately leading to a collapse and significant weight loss. In contrast, no immediate swelling was observed for the 23Gel-33, 99Gel-16, and 99Gel-33 formulations; however, gradual swelling was observed over time. These results demonstrated how variation in molecular weight and the rate of modification can affect the swelling behavior and the stability of the hydrogels. A lower degree of substitution (DS%) generally results in hydrogel networks with larger interchain distances, enhancing their capacity to absorb water. However, this relationship is influenced by the molecular mass distribution of the HASH chains, which can modulate the impact of DS% on water absorption.

#### 3.2.5 Hydrogels Redox-Stability Study

**Figure 9:**
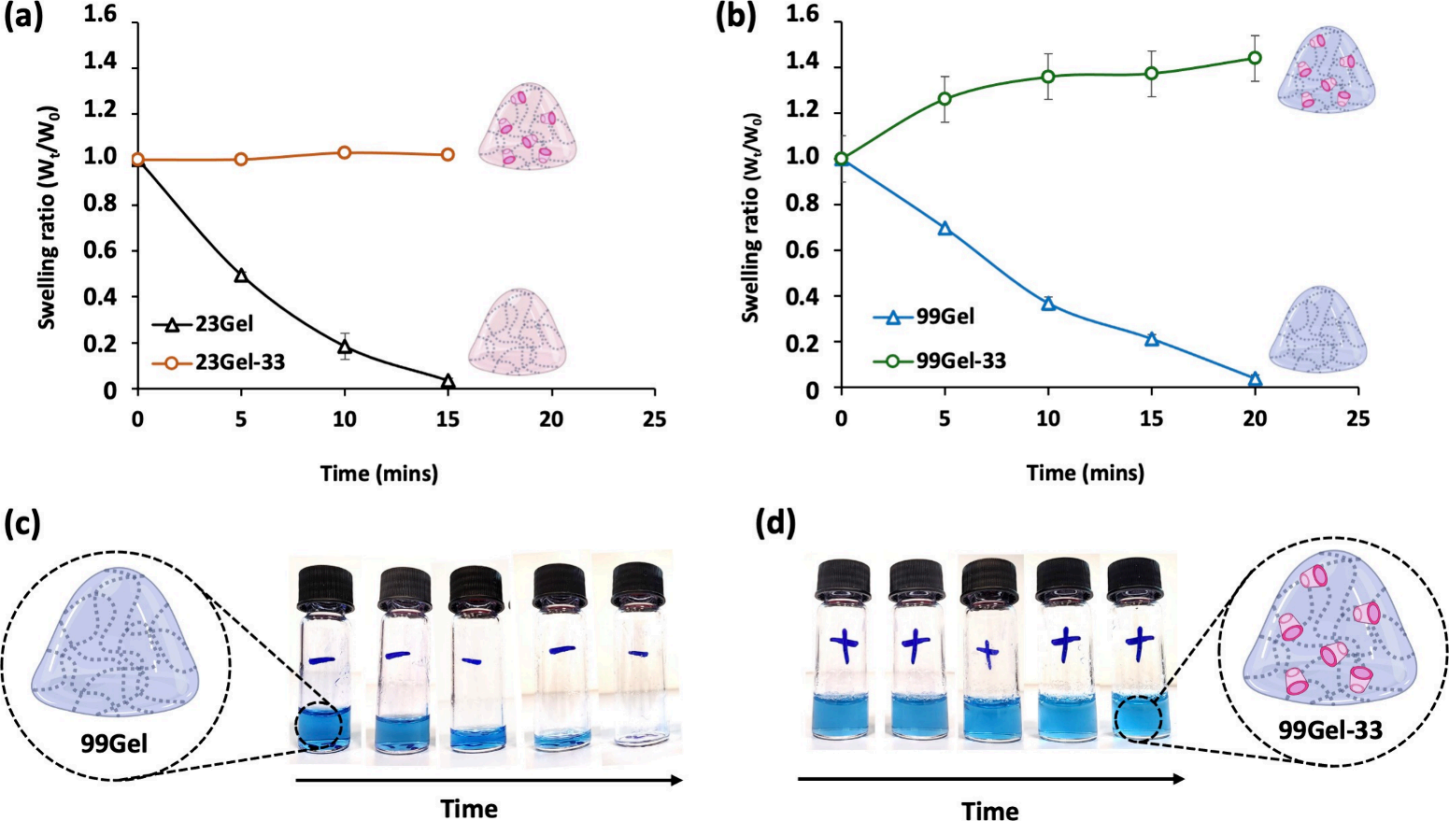
Hydrogels redox-stability test. (a) Swelling behavior over time of 23Gel vs 23Gel-33 when supplemented with a reducing agent as DTT (0.435 M); (b) swelling behavior over time of 99Gel vs 99Gel-33 when supplemented with 0.435 M DTT; (c) optical pictures of 99Gel when treated with 0.435 M DTT; and (d) optical pictures of 99Gel-33 when treated with 0.435 M DTT (mean ± SD, *n* = 2).

The thiol groups in HASH are highly prone to oxidation, leading to the formation of disulfide (−S–S−) bonds. Consequently, the presence of HASH introduces uncertainty regarding whether gel formation results from the intended Michael addition reaction or unintended disulfide bond formation. A redox-based stability and degradation test was conducted to clarify the cross-linking mechanism (Figure 9). The 23Gel-33 and 99Gel-33 formulations were investigated, including their respective 23Gel and 99Gel controls, where the CDVS cross-linkers were not added (hydrogel compositions described in Table 1). To mention, considering the faster gelation time of hydrogels with CDVS_33 than with CDVS_16, both the 23Gel-33 and 99Gel-33 hydrogels were used for this experiment. The selected hydrogels were supplemented with 0.435 M of DTT solution to mimic the reductive environment and were monitored regularly. DTT was chosen in this experiment particularly because of the high reductive sensitivity of the disulfide bond.^35^

As depicted in Figure 9, with each interval of 0.435 M DTT solution removal followed by the addition of fresh PBS supplemented with 0.435 M DTT, the weight of 23Gel and 99Gel lacking CDVS progressively decreased. In contrast, the 23Gel-33 (Figure 9a) and 99Gel-33 (Figure 9b) formulations containing CDVS maintained a stable weight over time, demonstrating their enhanced resistance to degradation under these conditions. That is because, as mentioned before, the disulfide bond is highly redox-sensitive and it undergoes breakage in the presence of a reducing agent, while the Michael addition bond will remain undisturbed. This proves that the 23Gel-33 and 99Gel-33 under study are being formulated successfully via the Michael addition bond. Additionally, preliminary observations during the hydrogel preparations showed a faster cross-linking time for the hydrogels with CDVS than those without CDVS. This can further substantiate the previous claim as the Michael addition cross-linking is typically faster than the disulfide bond formation. Furthermore, while 23Gel experienced complete network breakdown in 15 min, 99Gel took 20 min to degrade (Figure 9a,b). This indicates that hydrogels with lower molecular weight degrade more rapidly than those with higher molecular weight. Similar results were observed for 23Gel-16 and 99Gel-16, with optical images provided in Figure S2 of Supporting Information.

Overall, the study proved the stability of the Michael addition bond within the hydrogel network under a reductive environment. As part of maintaining cellular homeostasis and supporting various metabolic activities, reducing agents such as glutathione are prevalent in the physiological environment. This is especially true in diseased tissues, such as cancer, where the high metabolic rate of cells leads to elevated concentrations of reducing species.^65^ With these findings, the designed hydrogels promise their stability over time in physiological and diseased environments, ensuring controlled and sustained drug release.

#### 3.2.6 In Vitro Degradation Studies

Hydrogels, in general, can degrade through various mechanisms, including hydrolysis, oxidative processes, and enzymatic degradation, with each mechanism contributing differently based on the composition and environmental context of the hydrogel.^66^ When Sahoo *et al*.^67^ exhibited that HA-based hydrogels can degrade through enzymatic action or hydrolysis, with degradation rates influenced by cross-linking density and chemical modifications, Pang *et al.*(68) explained that gelatin-based hydrogels show resistance to hydrolytic degradation but degrade rapidly under oxidative and enzymatic conditions, with different pathways associated with the polypeptide backbone, urea linkages, and ester groups. Degradation can be achieved through dynamic physical bonds or reversible chemical cross-links, with factors such as reaction chemistry, cross-linking density, and network topology affecting the degradation profile.^69^

**Figure 10:**
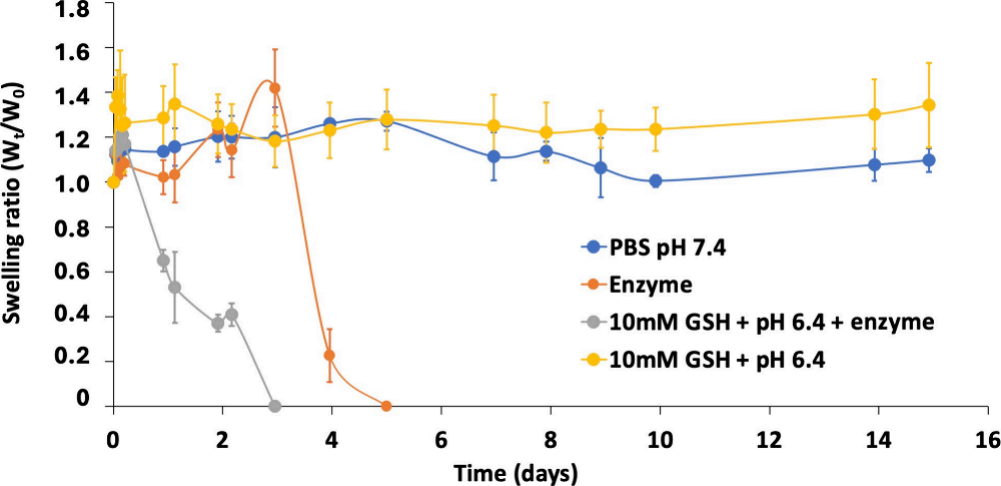
Swelling/degradation profiles of the 99Gel-33 hydrogel are shown as the variation in time under physiological, reductive, and enzymatic conditions at 37 °C (mean ± SD, *n* = 3).

The behavior of the 99Gel-33 hydrogels under study was observed when treated with different media to understand the influence of hydrolytic, enzymatic, and reductive action. As in Figure 10, the 99Gel-33 hydrogel was observed to be stable over time in both PBS pH 7.4 and 10 mM GSH + pH 6.4 media, proving gel stability in physiological conditions. A rapid weight loss due to enzymatic degradation was observed in both media containing the hyaluronidase enzyme. The fact that the 99Gel-33 hydrogels got degraded under enzymatic action is proof that the chemical modification of HASH did not affect its enzymatic degradability.^70^

Performing degradation studies is crucial for hydrogel-based systems as they directly influence functionality, safety, and performance, particularly in biomedical applications.^69^ They determine the hydrogel structural integrity and control drug release profiles. These studies also enable the customization of hydrogel properties to meet clinical requirements, such as tailored degradation rates and mechanical stability.^71^*In vitro* degradation mimics physiological conditions, providing baseline data on how the hydrogel will behave *in vivo*.^72^ Degradation studies provide essential insights by predicting *in vivo* behavior, guiding experimental design, reducing risks, and ensuring regulatory compliance, ultimately derisking *in vivo* testing and increasing the likelihood of clinical success.^73,74^ Based on the degradation study outcomes, the HASH/CDVS-based hydrogel under study is expected to remain stable under normal physiological conditions, supporting controlled and sustained drug delivery *in vivo*. The hydrogel susceptibility to enzymatic degradation, especially by hyaluronidases, indicates an effective breakdown in enzyme-rich tissues of tumor regions, facilitating drug release and clearance.

#### 3.2.7 *In Vitro* Cytocompatibility Assay

**Figure 11:**
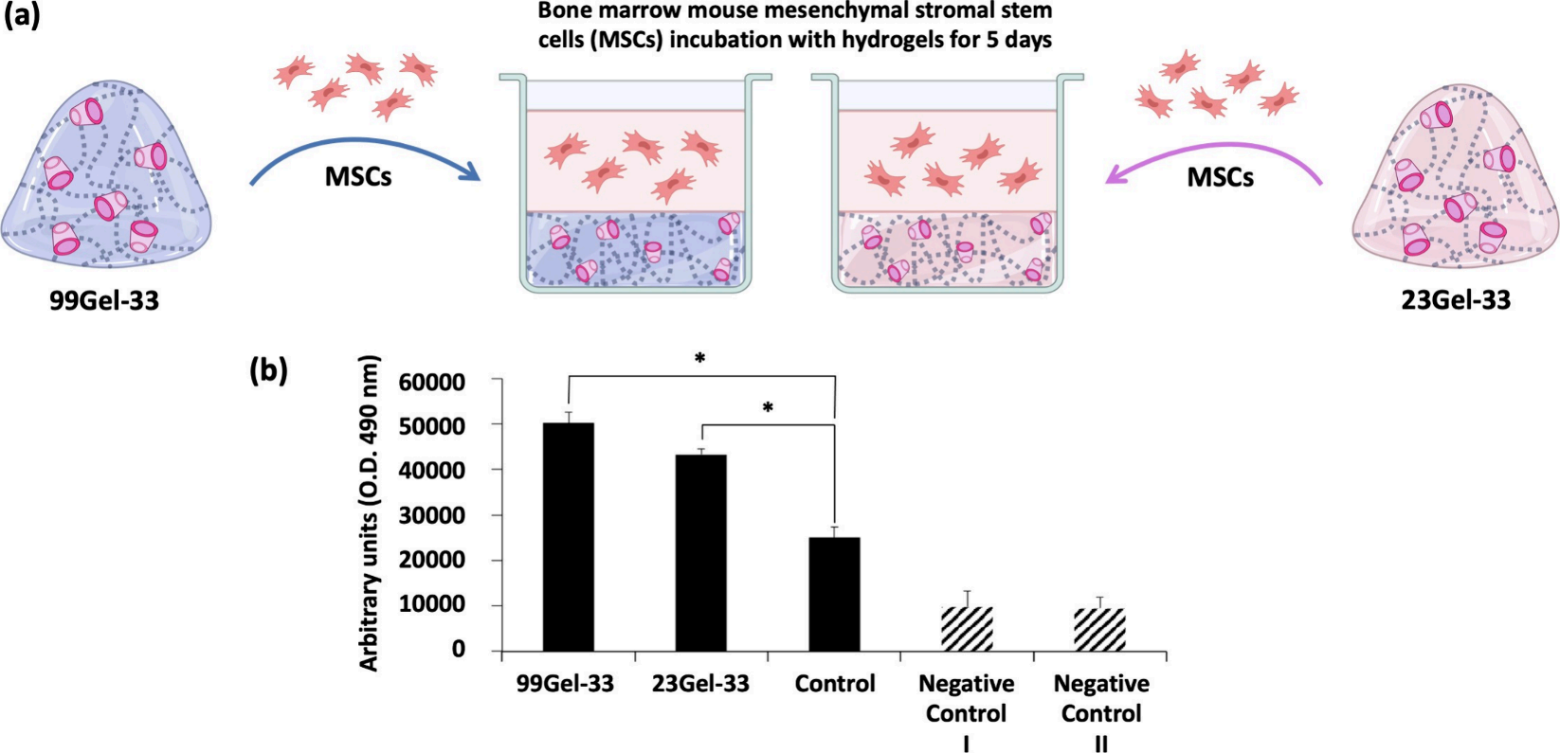
Cell viability data for mouse bone marrow-derived multipotent mesenchymal stem cells (MSCs) cultured on the 99Gel-33 and 23Gel-33 formulations after 5 days of incubation at 37 °C. (a) Schematic representation of the *in vitro* experiment. The Scheme was partially created with Biorender.com; (b) Significant increase of MSCs viability cultured with 99Gel-33 or 23Gel-33 compared with MSCs only culture (Ctrl = no treatment). Negative Ctrl l and negative Ctrl ll respectively represent the wells with hydrogels 99Gel-33 and 23Gel-33 alone. *p < 0.05.

To evaluate the physiological features of HA/CD-based hydrogels, MSCs were used for cell viability tests (Figure 11a). As shown in Figure 11b, both 99Gel-33 and 23Gel-33 hydrogels tested showed excellent biocompatibility. Interestingly, after 5 days of culture, an increase in MSC viability was found in the hydrogels-coated wells, suggesting an antiapoptotic/proliferating potential of the tested compounds. Research by Burdick and Prestwich. demonstrated that MSCs cultured in HA hydrogels exhibited higher viability and proliferation rates compared to those on traditional tissue culture plates.^75^ Wu *et al.* highlighted that HA hydrogels significantly reduced apoptosis in MSCs by providing a protective microenvironment.^76^ The current observation of increased cell viability and biocompatibility by 99Gel-33 and 23Gel-33 aligns with the findings above-mentioned. Furthermore, the enhanced cell proliferation observed can be attributed to the hydrophilic and biocompatible nature of HA as reported by Kim *et al*.^77^ The hydrophilic soft scaffold of HA hydrogel can mimic natural extracellular matrix (ECM), which reduces cellular stress and mechanical strain. This justifies the increased cell viability on 99Gel-33 compared to 23Gel-33 (Figure 11), as the former has a higher level of hydrophilicity. Indeed, MSCs are broadly appreciated in biomaterial research due to their regenerative/trophic potential, their anti-inflammatory role, and thus, their extensive therapeutic applications. MSCs serve as a standard model for assessing the biocompatibility and cytotoxicity of biomaterials, including hydrogels, as they can also interact with various components of the ECM.^78,79^ Additionally, the multipotent differentiation potential of MSCs, attributing to these cells a wide variety of applications such as bone repair, cartilage regeneration, and soft tissue engineering. Of interest, MSC-based systems are gaining traction in cancer treatment research, where hydrogels like HA/CD-based formulations may serve as drug delivery vehicles, tumor microenvironment mimics, or platforms for targeted therapy. The cell-friendly newly formed biomaterials proposed pave the way for novel, efficient therapeutic protocols in the field of drug delivery.

### 3.3 DOX/CRV-Loaded Hydrogels

The well-known ability of CDs and their derivatives to form stable inclusion complexes is widely exploited in the pharmaceutical field through supramolecular chemistry.^80^ This complex formation provides improved aqueous solubility and stability enhancement to the guest molecule, protecting it from degradation and allowing a controlled release profile.^81^ Considering its established antitumor properties, CRV was chosen in this study as the hydrophobic therapeutic to form the inclusion complex with CD. When Trindade *et al.*(82) demonstrated the potent antiproliferative effects against prostate cancer cells (PC3) *in vitro*, López *et al.*(83) highlighted the growth inhibition of CRV on colorectal cancer cells (HCT-116). Both papers discuss how the inclusion complexation of CRV with CD significantly enhanced its contribution to the cytotoxic effect on cancer cells. Additionally, Guimarães *et al*. successfully investigated the effectiveness of CRV when encapsulated in CD to enhance its antihyperalgesic effects, particularly in managing cancer pain.^84^ This study performed a simple and reproducible freeze-drying procedure for the CRV complexation with CDVS_33, keeping the same molar ratio between the complexing agent and the drug. Quantification of CRV within the CD cavity was done using GC for its high resolution and sensitivity, especially when coupled with MS, allowing the detection of lower concentrations.^85,86^ The obtained CDVS-CRV complex revealed an EE% of 38% with an LC of 5%.

**Figure 12:**
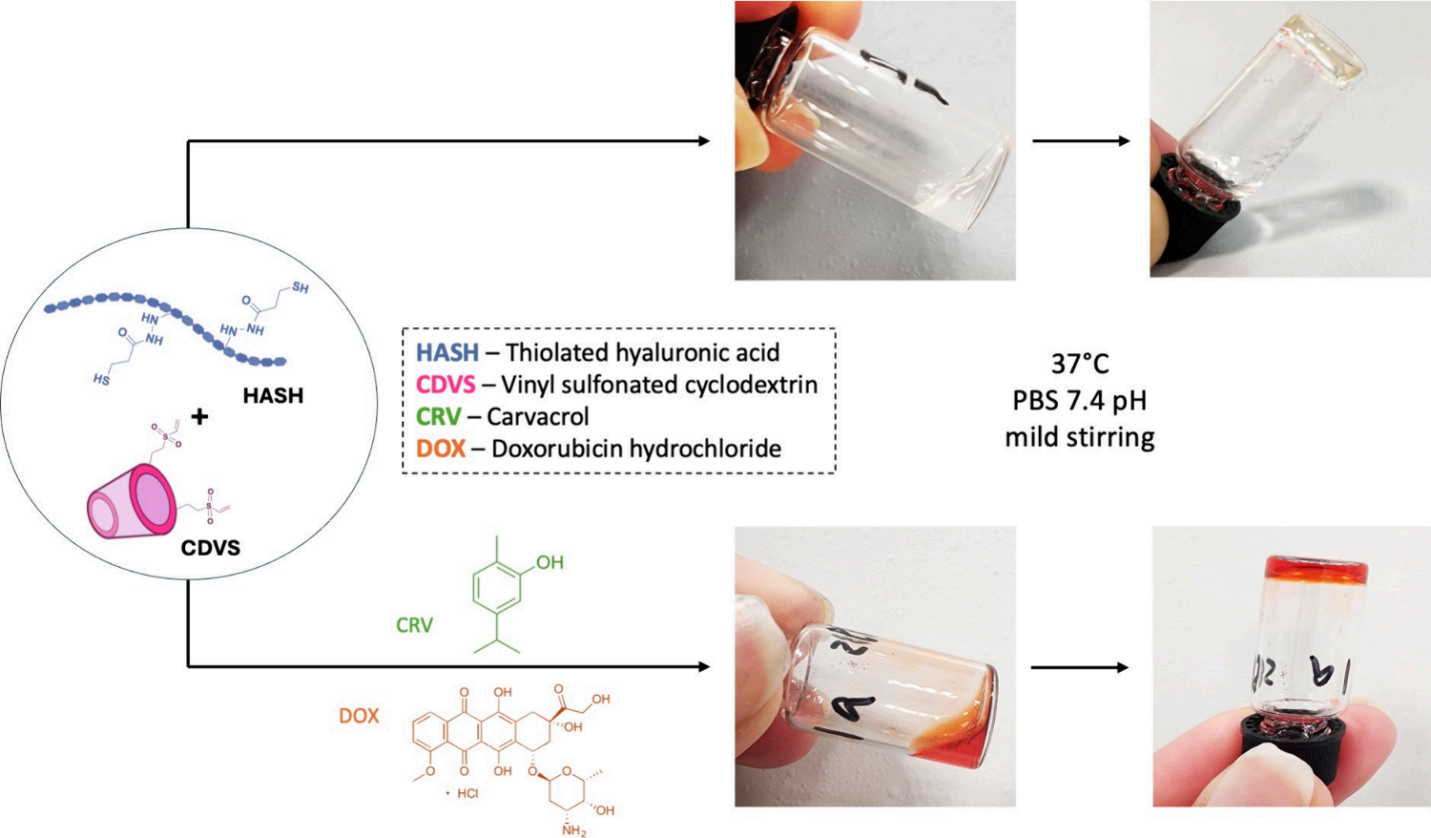
Pictorial representation of the formulation of the placebo 99Gel-33 hydrogel and 99Gel-33 hydrogel loaded with carvacrol and doxorubicin hydrochloride.

For the drug-loaded hydrogel, the CDVS-CRV complex was reacted with HASH-DOX under aqueous conditions to achieve the final formation of 99Gel-33, serving as an ideal platform for these studies. While CRV was incorporated within the CD cavity via host–guest inclusion complexation by noncovalent interaction before hydrogel preparation, DOX was added during the gelation process along with 99-HASH due to its hydrophilicity. DOX is used as the model hydrophilic agent due to its widely known potent antitumor effects and combinatorial therapy efficacy.^87^ Researchers have successfully demonstrated its potential in treating breast cancer and other solid tumors.^88^ The rapid gelation ensures localized hydrogel formation at the target site in a minimally invasive manner while keeping the hydrogel system chemically stable and can be engineered for tunable degradation rates. This process can occur under mild conditions, requiring no harsh catalysts, allowing it to be exploited to carry cocktails of sensitive drugs. A study by Peterson *et al.* developed a targeted polymersomes-based system using amphiphilic diblock copolymer, where thiol–ene Michael addition reaction was applied for attaching targeting peptides to the polymersomes. The vinyl sulfone electrophile reacted specifically with thiol groups, ensuring that only peptides with thiol functionality are modified, minimizing unwanted side reactions. The study further demonstrated the efficiency of the reaction in enhancing system specificity under mild conditions.^89^Figure 12 shows the pictorial demonstration of the formulation of both placebo and CRV/DOX-loaded 99Gel-33 hydrogels.

#### 3.3.1 *In Vitro* Release Studies

The simultaneous *in vitro* release behavior of both DOX and CRV from the developed 99Gel-33 hydrogel system was studied in physiological and tumor-resembling conditions. DOX and CRV were chosen for this study due to their complementary therapeutic properties, as DOX is a potential chemotherapeutic agent that intercalates DNA and inhibits topoisomerase II causing cancer cell apoptosis, while CRV is a natural compound that possesses anti-inflammatory and antioxidant as well as anticancer properties enhancing DOX efficacy while possibly reducing its side effects.^84,88^ This combination allows for a synergistic effect, potentially improving treatment outcomes while utilizing the hydrogel system for localized drug release.

**Figure 13:**
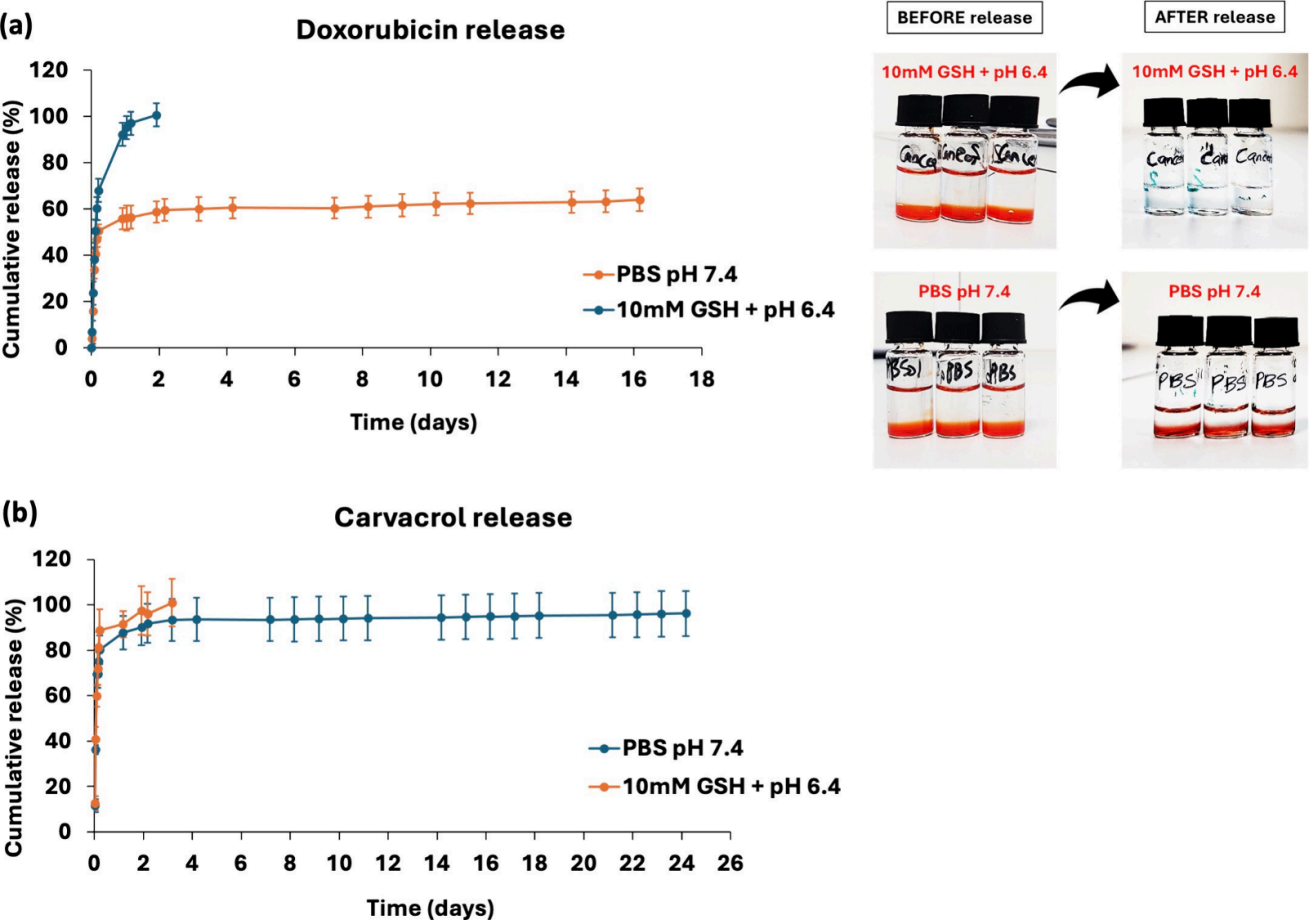
(a) DOX cumulative release profile % as a function of time analyzed using a fluorometer along with images of loaded 99Gel-33 hydrogels before and after release visually demonstrating the DOX release; (b) CRV cumulative release profile % as a function of time obtained by GC analysis (mean ± SD, *n* = 3).

While monitoring the release profile of hydrophilic DOX in physiological (PBS pH 7.4) and tumor-mimicking (10 mM GSH + pH 6.4) media (Figure 13a), there was an initial burst release of about 50.6 and 68.1% in the first 5 h. The higher release rate observed in the cancer media eventually reached 100% within 2 days (46 h), whereas only 58.7% of release occurred in physiological conditions by that time. The initial rapid drug release can be beneficial in treating cancer cells, where it requires immediate action, as stated by Han *et al*., who displayed maximized tumor inhibition and metastasis by delivering a high concentration of the drug to tumor sites through the initial sudden drug release from the HA-based hydrogel system.^90^ Over the course of 16 days, the cumulative DOX release reached only approximately 64% in the physiological condition. This slow release could be due to the charge interaction between the negatively charged HA and positively charged DOX. This drug retention allows its accumulation in tumor tissue, followed by stable and prolonged treatment quality.^91,92^ A negatively charged hydrogel system developed in a study by Dadsetan *et al*., which effectively loaded positively charged DOX through ionic bonding, demonstrated controlled release due to DOX retention that minimized the peak concentrations of the drug, potentially reducing side effects associated with high doses of free drug, and preserves antitumor efficacy.^93^ The images of 99Gel-33 hydrogel (Figure 13a) visually display that after the release experiments, DOX (indicated by the orange color) is completely released in cancer-mimicking media, while some of the drug is still retained in hydrogels treated with PBS pH 7.4 media.

Simultaneously, in the case of CRV, in both PBS pH 7.4 and 10 mM GSH + pH 6.4 media, an initial burst of about 80.2 and 88.7% was observed, respectively, in 5 h. A 100% release was observed under tumor-mimicking conditions after 3 days (76 h), while 93.3% happened in the physiological state at that same time. From the third day up to 24 days, the system exhibited a slow sustained release from 93.3 to 96.2% in the physiological conditions as shown in Figure 13b. This slow controlled release retains the therapeutic level for a longer duration, as demonstrated by Kim *et al*.^94^ In a previous study by Fiorica *et al*.,^49^ only 50% of drug release occurred from the CD cavity even after 32 days of incubation since the host–guest hydrophobic interaction between the drug and the CD cavity causes significant retention of the drug in the hydrogel system, slowing down its diffusive release. However, in the current study, a higher release rate was observed for CRV (from the CD cavity) compared to DOX in the physiological media.

#### 3.3.2 DOX-Hydrogel Interaction

**Figure 14:**
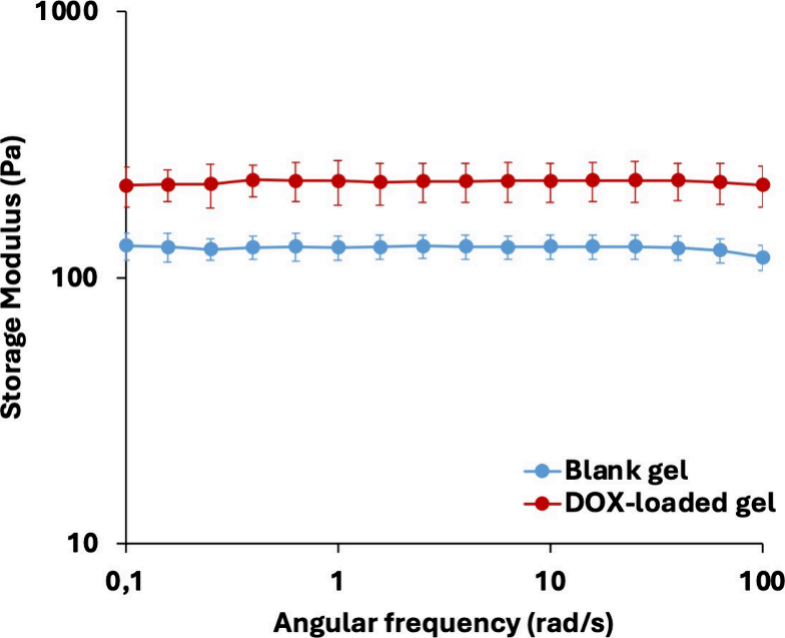
Rheological measurement showing the storage modulus of 99Gel-33 blank and DOX-loaded 99Gel-33 hydrogels as a function of angular frequency in rad/s (mean ± SD, *n* = 2).

A rheological analysis involving the frequency test was performed on 99Gel-33 blank and DOX-loaded 99Gel-33 hydrogels to understand the slow release of DOX observed in section 3.3.1 (Figure 13a). In rheology, an increase in *G*′ (storage modulus indicating the gel strength) after incorporating charged molecules suggests enhanced cross-linking or interactions, such as ionic bonding, between the hydrogel matrix and the incorporated species.^95^ In a previous study, the role of ionic interactions was demonstrated with increased *G*′ in hydrogels loaded with positively charged drugs like vancomycin. This increase indicated that vancomycin acted as a cross-linker, forming ionic bonds between its amine groups and the negatively charged carboxyl groups of anionic polysaccharides, such as hyaluronic acid and xanthan gum.^55^ Similarly, in the current study, DOX-loaded hydrogel exhibits higher storage modulus values than the blank hydrogel throughout varying angular frequencies, as displayed in Figure 14. For example, at an angular frequency of 40 rad/s, the *G*′ value of the hydrogel increased from 129.5 to 232.4 Pa upon loading DOX, indicating an increase in the strength of the hydrogel network. This proves a charge-based bond formation within the hydrogel, due to the ionic interactions between positively charged DOX (specifically the primary amine group on its daunosamine sugar) with the negatively charged carboxyl group of HA polymer, providing enhanced gel elasticity.

### 3.4 *In Vitro* Antitumor Activity

#### 3.4.1 MTT Assay

**Figure 15:**
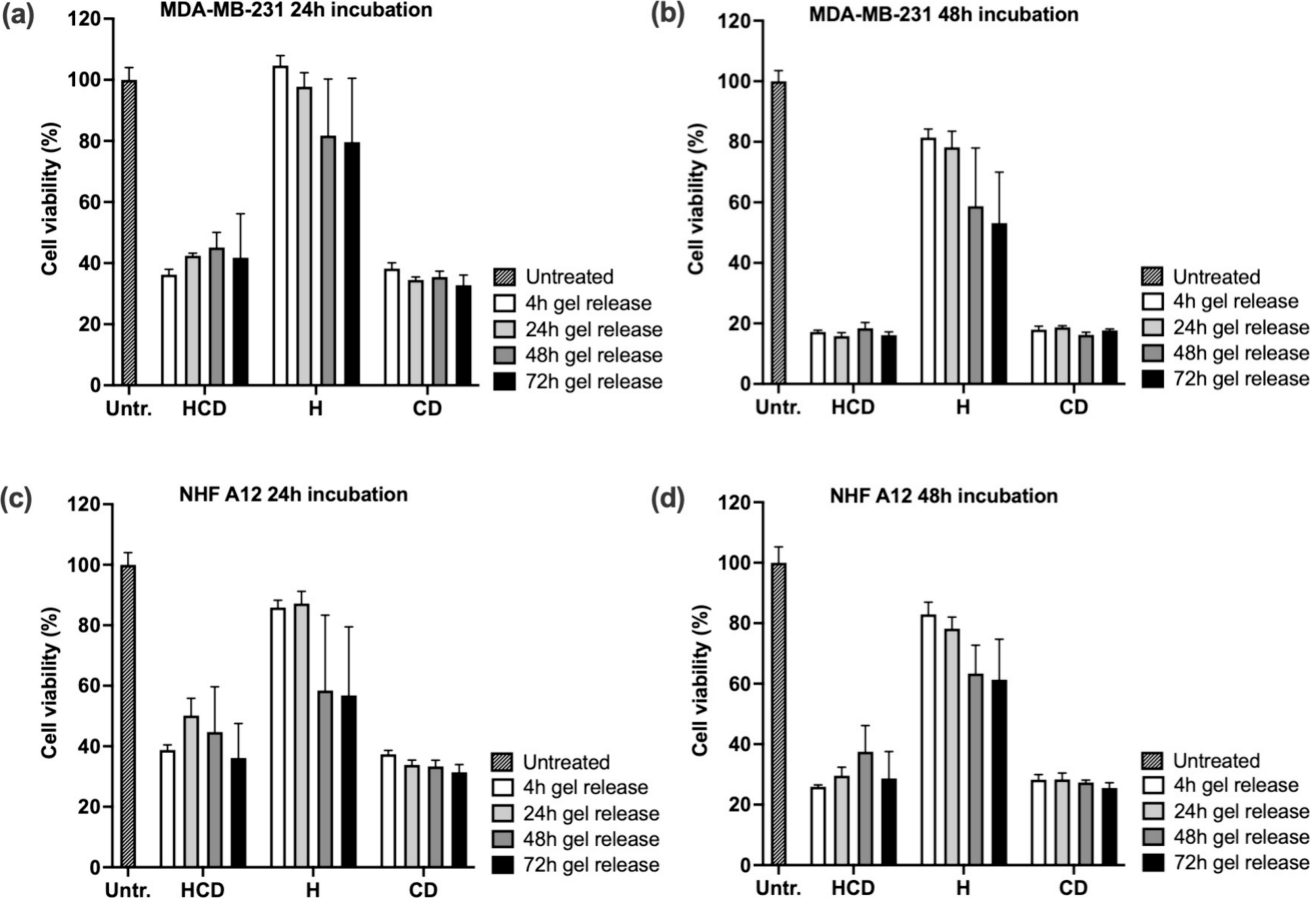
MTT cell viability assay of MDA-MB-231 triple-negative breast cancer cells upon (a) 24 h and (b) 48 h incubation and NHF A12 primary human stabilized fibroblast cell lines upon (c) 24 h and (d) 48 h incubation, mean ± SD, *n* = 3 (HCD = DOX/CRV-loaded 99Gel-33 hydrogel; H = placebo 99Gel-33 hydrogel; CD = free CRV and DOX drugs combined; and untreated = placebo cells as control).

To assess the antitumor efficacy and cytocompatibility, the MDA-MB-231 triple-negative breast cancer cell lines and the NHF A12 primary human stabilized fibroblast cell lines were incubated with DOX/CRV-loaded hydrogel and free DOX/CRV drugs. Cell viability was assessed using the MTT assay after 24 and 48 h of incubation based on the drug release (section 3.3.1) at desired time points of 4, 24, 48, and 72 h, as revealed in Figure 15. For MDA-MB-231 cancer cells, the DOX/CRV-loaded 99Gel-33 hydrogel significantly reduced cell viability across all time points, indicating its potent antitumor effect. The free DOX/CRV drugs alone showed a similar reduction in viability, confirming their inherent cytotoxicity against the cancer cells, while the placebo hydrogel exhibited minimal effect. With NHF A12 fibroblast, the placebo hydrogel showed excellent cytocompatibility with minimal impact on normal cell viability. Zhao *et al.* highlighted that the hydrogels designed with natural polymers (e.g., alginate, hyaluronic acid) exhibited minimal effects on fibroblast viability, evidencing their safety as carriers.^96^ Alternatively, hydrogels incorporating targeted drugs, as reported by Li *et al*.,^97^ induced apoptosis in breast cancer cells and reduced tumor progression in preclinical models, emphasizing the potential of hydrogel systems for localized and effective cancer therapy. The results obtained in this study underline the potential of the DOX/CRV-loaded 99Gel-33 hydrogel system as an efficient delivery platform for cancer therapy, by retaining the drug antitumor activity with controlled release properties. Studies have stated that controlled-release systems can improve drug efficacy by maintaining drug concentrations in the therapeutic window, reducing drug resistance, and enhancing apoptosis in cancer cells.^98^ The prolonged reduction in cancer cell viability across 4 to 72 h indicates effective drug release and action by the loaded system, consistent with this principle. However, the moderate cytotoxicity observed in NHF A12 fibroblasts indicates the need for optimization of the drug dosage or hydrogel composition to enhance selectivity and minimize adverse effects on healthy cells. Overall, the DOX/CRV-loaded 99Gel-33 hydrogel system demonstrates promising therapeutic potential, with the sustained reduction in cancer cell viability over time reflecting its capability for prolonged and effective drug release, warranting further investigation into its long-term effects and mechanisms of action.

#### 3.4.2 Drug Uptake in MDA-MB 231

**Figure 16:**
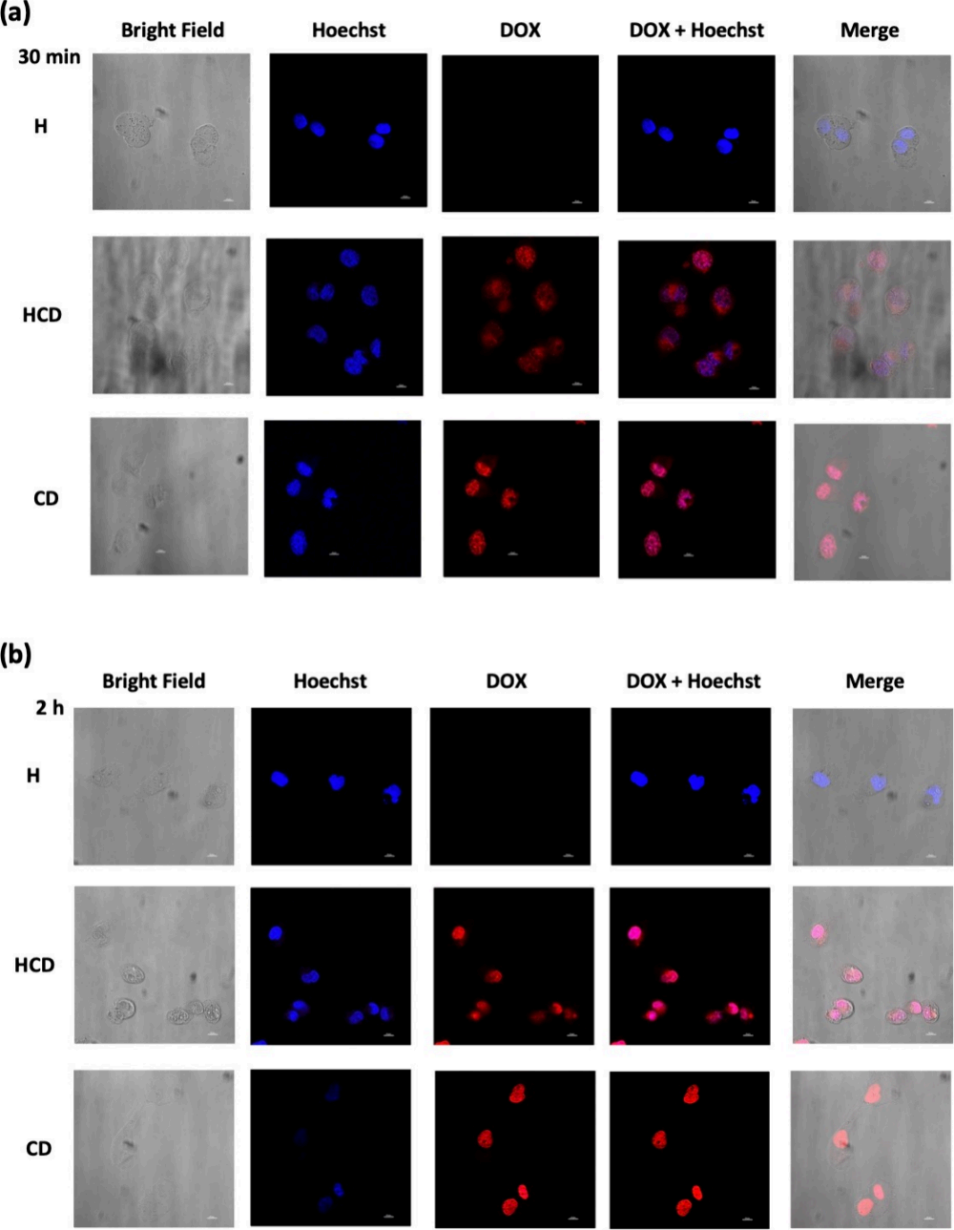
Uptake by MDA-MB 231 cells of DOX after 30 min (a) and 2 h (b) incubation with free DC and DC-loaded 99Gel-33 hydrogels, visualized by confocal microscopy. Nuclei were stained with Hoechst 33324 (HCD = DOX/CRV-loaded 99Gel-33 hydrogel; H = placebo 99Gel-33 hydrogel; CD = free CRV and DOX drugs combined; Untreated = placebo cells as control).

The time-dependent uptake and localization of DOX/CRV released from the DOX/CRV-loaded 99Gel-33 hydrogel were investigated by incubating the loaded delivery systems with MDA-MB 231 cells and followed by confocal microscopy. Uptake patterns are shown in Figure 16. After 30 min of incubation with free drugs, red fluorescence was observed in cell nuclei, suggesting the rapid intercalation of intracellular DOX to chromosomal DNA after passive diffusion into the cells (Figure 16a), and the fluorescence intensity increased significantly after 2 h post-treatment (Figure 16b). Similarly, it was evidenced that in the cells treated with drug-loaded hydrogel, the red fluorescence was mainly located in the nucleus already after 30 min incubation, suggesting that the entrapped drug was released from the hydrogel system and uptake as a free drug, as shown by cells incubated with free drugs. A previous study where MCF-7 cancer cells were cultured with various concentrations of DOX-loaded hydrogel reported effective internalization of the drug by the cells after 4 and 24 h, with a notable increase in uptake at higher concentrations of DOX.^99^ Research by Gallo *et al.* developed peptide-based hydrogels, reporting the effective encapsulation of DOX within the hydrogels, and demonstrated a significant reduction in cell viability of MDA-MB-231 cells after 24 h of incubation, indicative of effective drug uptake.^100^ Furthermore, the enhanced intracellular distribution of DOX in MDA-MB-231 by the use of functional exosome-mediated codelivery systems was evidenced in another study using confocal microscopy.^101^ These studies collectively align with the findings obtained in the current study. The overlay images in Figure 16 confirm the colocalization of the drugs within the nuclei, with stronger signals over time, reinforcing the potential of the DOX/CRV-loaded hydrogel system for improved intracellular drug accumulation.

#### 3.4.3 Annexin V Assay

**Figure 17:**
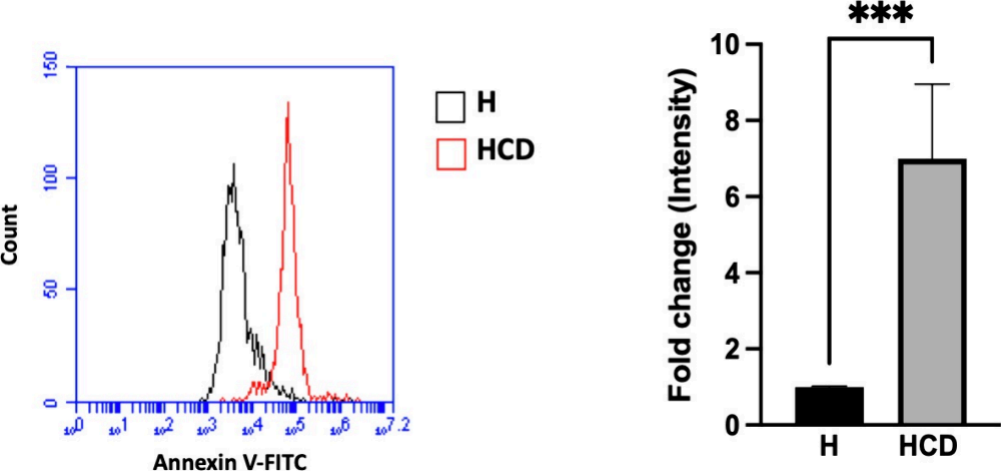
Cell death was evaluated by Annexin V-FITC staining and flow cytometric analysis. The MFI values of HCD cells were normalized to those of the H cells. ***p < 0.001, mean ± SD, *n* = 3 (H = placebo 99Gel-33 hydrogel; HCD = DOX/CRV-loaded 99Gel-33 hydrogel).

The apoptosis of the MDA-MB 231 cells upon treatment with placebo 99Gel-33 hydrogel and drug-loaded 99Gel-33 hydrogel was investigated through the Annexin V staining assay. In this assay, a rightward shift in fluorescence on the graph typically indicates an increase in apoptotic cells.^102^ The minimal fluorescence shift by placebo hydrogel, as observed in Figure 17, indicates that it does not significantly affect the apoptotic status of the cancer cells, highlighting its biocompatibility as a delivery platform, as it does not trigger unwanted cell death. Conversely, a pronounced right shift in the fluorescence was observed by the loaded hydrogel system after 2 h of treatment, demonstrating the activation of the cell death pathway due to the action of dual-drug delivery. This suggests efficient drug release from hydrogel and effective interaction with cancer cells, causing apoptosis. The key biological significance of this study is the rapid activation of apoptosis, observed within just 2 h of treatment with the drug-loaded hydrogel, demonstrating that the released drug swiftly disrupts cancer cell survival mechanisms. A previous study investigated a pH-responsive hydrogel that codelivered DOX with Conferone (Conf) to MDA-MB-231 cells, where the results showed that this combination therapy significantly increased apoptosis rates, with over 98% of the cell population undergoing apoptosis at a specific concentration, and Annexin V analysis confirmed the effectiveness of the drug-loaded hydrogel in inducing cell death compared to placebo hydrogels.^103^ The fold change in fluorescence intensity in the Annexin V-FITC assay reflects a relative increase in apoptotic cell populations. As observed in Figure 17, a minimal fold change for the placebo hydrogel suggests its safety, while a significant fold change of about 60% more for the drug-loaded hydrogel, indicating robust Annexin V binding, highlights its potent anticancer activity through apoptosis. This fold change serves as a key metric crucial for evaluating the therapeutic effectiveness and selectivity of drug delivery systems.

## 4 Conclusions

A tunable in situ forming hydrogel system based on thiol–ene Michael addition conjugation between thiol-modified hyaluronic acid (HASH) and vinyl sulfone-functionalized β-cyclodextrin (CDVS) was developed for localized cancer therapy. For the first time, this work investigated the tailorable properties and dual-drug delivery approach of the HASH/CDVS system using hydrophilic (DOX) and hydrophobic (CRV) therapeutics. HASH molecular weight and CDVS modification influenced hydrogel properties, with 99Gel-33 showing superior viscoelasticity. The hydrogel proved to be stable physiologically, enzymatically degradable, and biocompatible, promoting stem cell growth. Distinct DOX/CRV release kinetics enabled immediate and sustained effects, enhancing cancer treatment efficacy. In conclusion, the findings highlight the versatility of this platform, demonstrating that straightforward modifications in molecular weight and precursor functionality allow precise tuning of mechanical properties, positioning it as a promising tool for a wide range of biological applications. This innovative approach emphasizes the potential of hydrogels in personalized combinatorial cancer therapy and opens possibilities for further clinical translation and optimization.

## Supporting Information Available

Additional experimental details for the study on localized cancer treatment using thiol–ene hydrogels for dual drug delivery. It includes Fourier-Transform Infrared Spectroscopy (FTIR) analysis to characterize the chemical composition of the hydrogels, mechanical characterization through rheology measurements to determine mesh size and cross-linking density, a redox-stability study analyzing degradation and swelling behavior, and thermal analysis using DSC and TGA (PDF)
