# Supplementary material for: Localized Cancer Treatment Using Thiol–Ene Hydrogels for Dual Drug Delivery
Source: Biomacromolecules. 2025 Apr 8;26(5):3234–54. doi: 10.1021/acs.biomac.5c00387 (PMC12076507; doi:10.1021/acs.biomac.5c00387)
Supplement: Supplementary file 1 — bm5c00387_si_001.pdf [file bm5c00387_si_001.pdf]

Supporting Information

Localized Cancer Treatment Using Thiol-Ene Hydrogel for Dual Drug Delivery

Lakshmi Sathi Devi[1], *Maria Rosa Gigliobianco*[2], *Serena Gabrielli*[3], *Dimitrios Agas*[4], *Maria Giovanna Sabbieti*[4], *Maria Beatrice Morelli*[5], *Consuelo Amantini*[4], *Cristina Casadidio*[1,*], *Piera Di Martino*[2] *and Roberta Censi*[1]

[1]School of Pharmacy, University of Camerino, ChIP Chemistry Interdisciplinary Project Research Centre, Via Madonna delle Carceri, 62032 Camerino (MC), Italy

[2]Department of Pharmacy, University of "G. D'Annunzio" Chieti and Pescara, Via dei Vestini 1, 66100 Chieti (CH), Italy

[3]School of Science and Technology, University of Camerino, ChIP Chemistry Interdisciplinary Project Research Centre, Via Madonna delle Carceri, 62032, Camerino (MC), Italy

[4]School of Biosciences and Veterinary Medicine, University of Camerino, Via Gentile III da Varano, 62032 Camerino (MC), Italy

[5]School of Pharmacy, University of Camerino, Department of Experimental Medicine and Public Health, Via Madonna delle Carceri, 62032 Camerino (MC), Italy

*Corresponding Author: Dr. Cristina Casadidio,
School of Pharmacy,
University of Camerino,
ChIP Chemistry Interdisciplinary Project Research Centre, Via Madonna delle Carceri, 62032
Camerino (MC), Italy.
Email: cristina.casadidio@unicam.it

S1. Fourier-Transform Infrared Spectrophotometer (FTIR)

The chemical composition of the polymers and hydrogels was studied using an attenuated total reflectance Fourier-transform infrared spectrophotometer (FTIR, PerkinElmer, US) at a wavelength range of 4000-400 cm^{-1} by comparing the infrared spectra of CDVS_33, 99-HASH, and lyophilized 99Gel-33 (Figure S1). The spectral analysis reveals significant similarities between the signals of 99Gel-33 and 99-HASH, indicating comparable structural or functional characteristics between the two samples. Indeed, the 99Gel-33 and 99-HASH showed the major characteristic peak of stretching vibrations of O-H and N-H bonds around 3260 cm^{-1} [1], and CDVS_33 showed the symmetric and asymmetric stretching vibrations of O-H groups at 3344.10 cm^{-1} [2]. Around 2925 cm^{-1}, all three spectra showed the stretching vibrations of their corresponding C-H bond. Additionally, the signals at 1614.4 and 1414.3 cm^{-1} of 99-HASH attributed to the symmetric C=O vibrations of N-acetyl and asymmetric C=O stretching of carboxylate groups of the HA chain can also be observed in the 99Gel-33 spectrum [1, 3]. Furthermore, CDVS_33, 99-HASH, and 99Gel-33 showed absorption signals at around 975-1169 cm^{-1} corresponding to the C-O-C and C-O stretching vibration of their glycosidic ether bond and primary alcohol groups [1, 2, 3]. CDVS_33 also showed the H-O-H bending vibration of water present at 1658.55 cm^{-1} [4, 5]. As expected by the feed ratio of the polymers, these results confirm the 99Gel-33 composition with a high share of 99-HASH and a relatively low share of CDVS_33. Although the FTIR outcome reveals the successful presence of both CD and HA in the hydrogel composition, the more relevant peaks such as of thiols, vinyl sulfones, and Michael addition bond required to comment on the crosslinks were not observed, possibly because of their poor absorption intensity [3, 6].

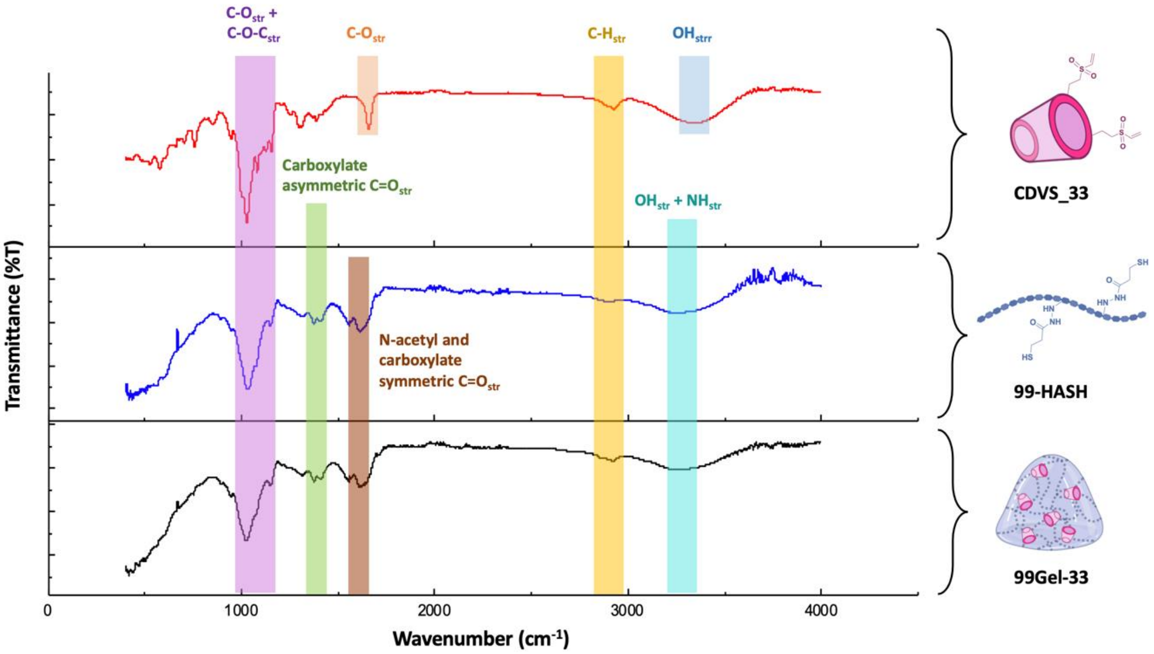

Figure S1. FTIR spectra of CDVS_33, 99-HASH, and 99Gel-33 between 400 and 4000 cm^{-1}. The measurements were performed on dried samples.

S2. Hydrogels mechanical characterization

Rheology analyses were employed to estimate the average mesh size (ξ) of hydrogels in their hydrated state. The mesh size, measured in nanometers (nm), represents the distance between crosslinking points within the polymer network [7]. This parameter can be determined using rubber elastic theory (RET) with the following Equation S1:

$$\xi = \left(\frac{G'N_A}{RT}\right)^{-1/3} \qquad \textit{Equation S1}$$

where *G'* is the storage modulus, *R* represents the universal gas constant (8.314 J/K mol), *T* is the absolute temperature (310K) and N_A denotes Avogadro constant (6.022 x 10^{23}) [8, 9].

Another important structural property of hydrogels is the crosslinking density (n_e) which indicates the number of elastically active junctions per unit volume (mol/m³). This parameter can also be derived using RET, as shown in the Equation S2 below:

$$n_e = \frac{G_e}{RT} \qquad \textit{Equation S2}$$

where G_e corresponds to the plateau storage modulus obtained from frequency sweep tests [10].

S3. Hydrogels redox-stability study

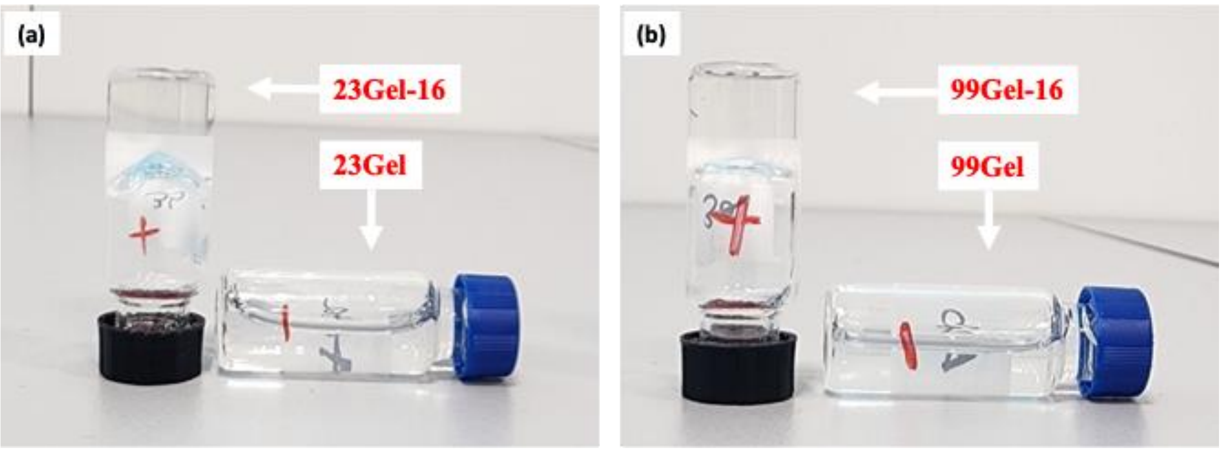

Figure S2. (a) Degradation behavior of 23Gel vs 23Gel-16 when supplemented with a reducing agent as DTT (0.435M DTT) after 20 minutes of incubation; (b) swelling behavior over time of 99Gel vs 99Gel-16 when supplemented with 0.435M DTT after 20 minutes of incubation.

S4. Polymers and hydrogels thermal analysis

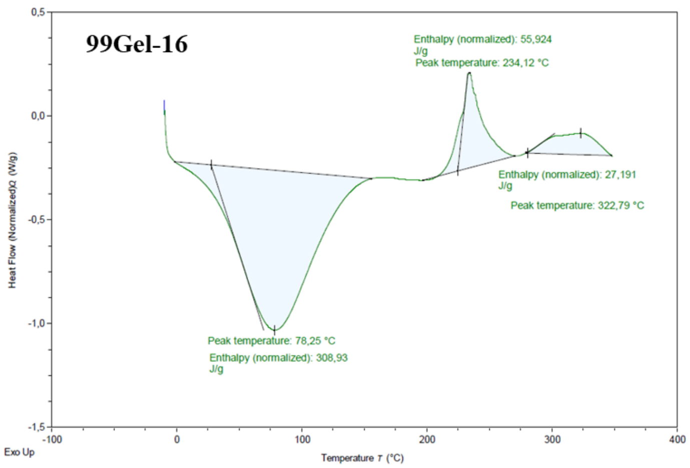

Figure S3. DSC trace of dried 99Gel-16 sample.

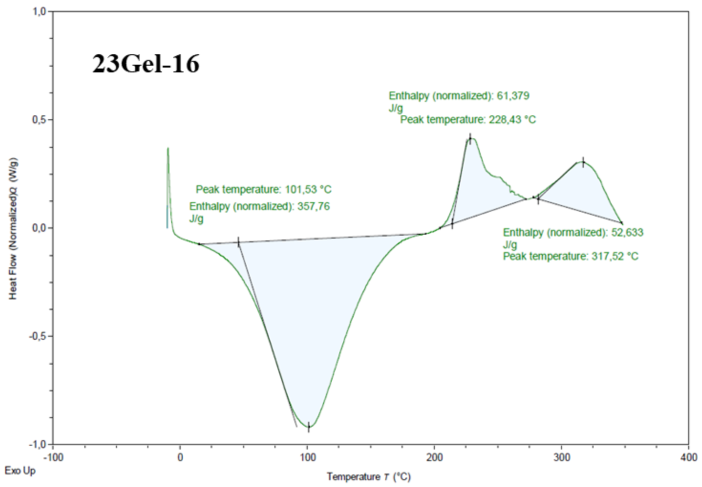

Figure S4. DSC trace of dried 23Gel-16 sample.

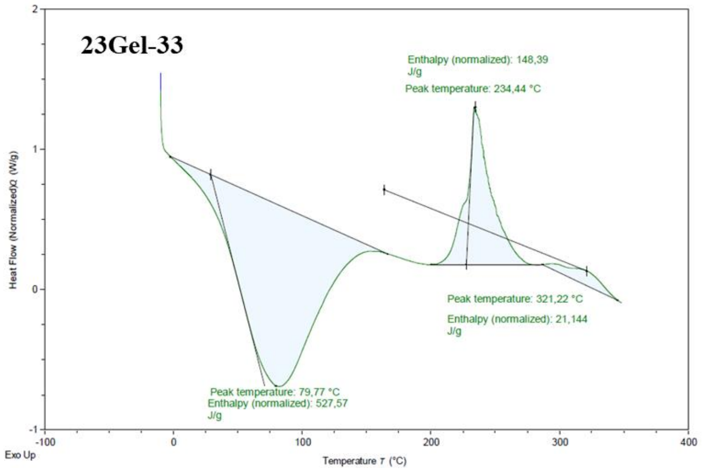

Figure S5. DSC trace of dried 23Gel-33 sample.

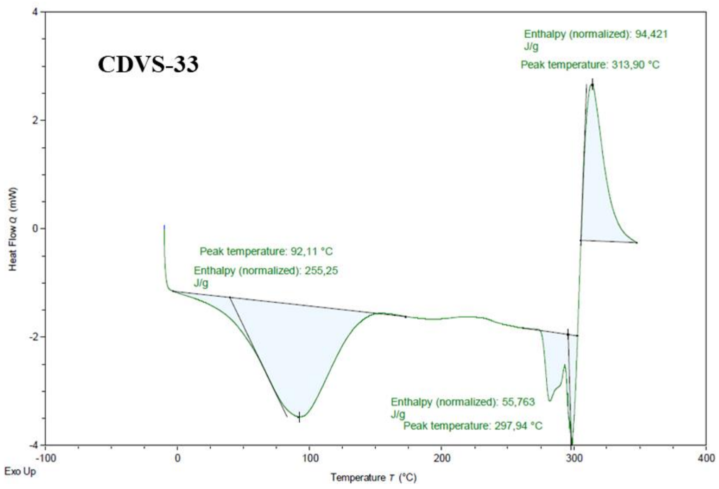

Figure S6. DSC trace of dried CDVS_33.

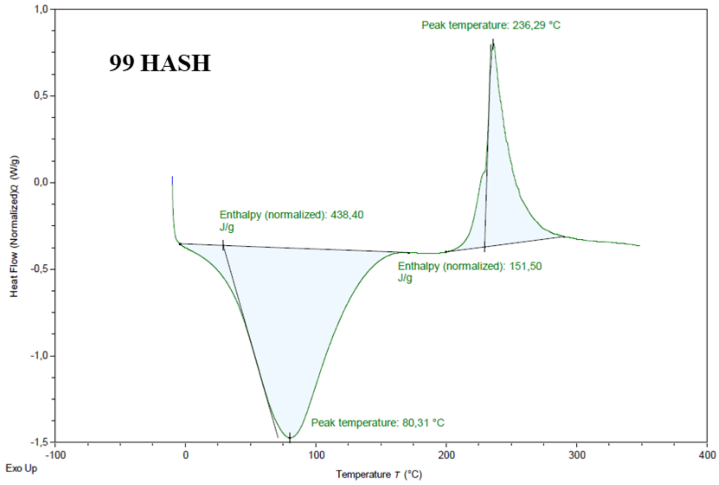

Figure S7. DSC trace of dried 99-HASH polymer.

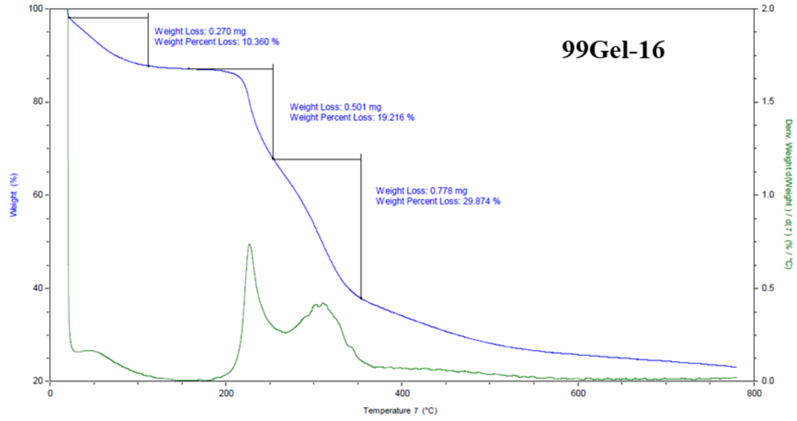

Figure S8. TGA trace of dried 99Gel-16 sample.

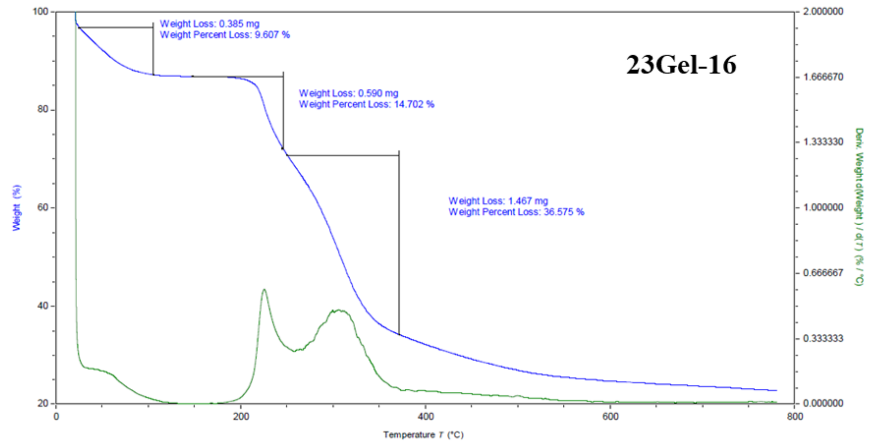

Figure S9. TGA trace of dried 23Gel-16 sample.

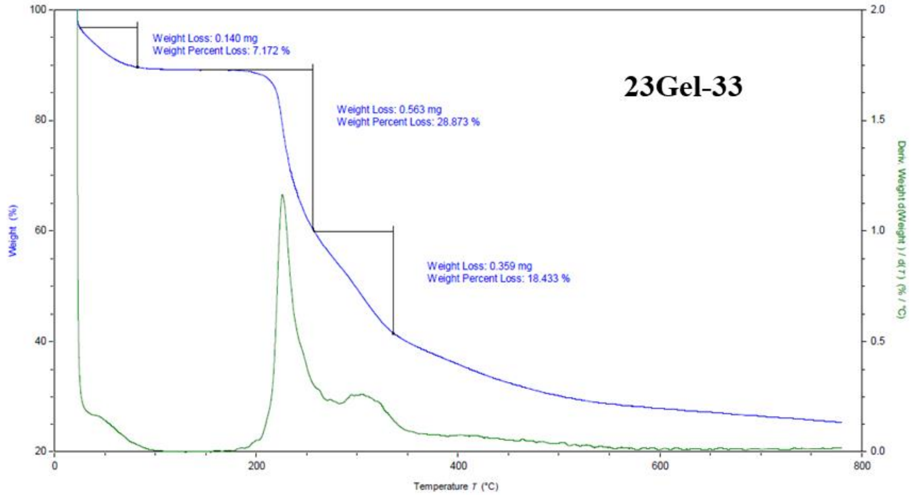

Figure S10. TGA trace of dried 23Gel-33 sample.

References

[1] J. Andrade del Olmo, J.M. Alonso, V. Sáez Martínez, L. Ruiz-Rubio, R. Pérez González, J.L. Vilas-Vilela, L. Pérez-Álvarez, "Biocompatible hyaluronic acid-divinyl sulfone injectable hydrogels for sustained drug release with enhanced antibacterial properties against Staphylococcus aureus," *Materials Science and Engineering: C,* vol. 125, p. 112102, 2021.

[2] A. Mahmood, M. Ahmad, R.M. Sarfraz, M.U. Minhas, "b-CD based hydrogel microparticulate system to improve thesolubility of acyclovir: Optimization through in-vitro,in-vivo andtoxicological evaluation," *Journal of Drug Delivery Science and Technology,* vol. 36, pp. 75-88, 2016.

[3] S. Deng, M.R. Gigliobianco, E. Mijit, M. Minicucci, M. Cortese, B. Campisi, D. Voinovich, M. Battistelli, S. Salucci, P. Gobbi, G. Lupidi, G. Zambito, L- Mezzanotte, R. Censi, P. Di Martino, "Dually Cross-Linked Core-Shell Structure Nanohydrogel with Redox–Responsive Degradability for Intracellular Delivery," *Pharmaceutics,* vol. 13, no. 12, p. 2048, 2021.

[4] S. Amani, A. Bagheri Garmarudi, N. Rahmani, M. Khanmohammadi, "The β-cyclodextrin-modified nanosized ZSM-5 zeolite as a carrier for curcumin," *RSC Adv.,* vol. 9, pp. 32348-32356, 2019.

[5] H. Rachmawati, C. Ariani Edityaningrum, R. Mauludin, "Molecular Inclusion Complex of Curcumin–β-Cyclodextrin Nanoparticleto Enhance Curcumin Skin Permeability from Hydrophilic Matrix Gel," *AAPS PharmSciTech,* vol. 14, no. 3, 2013.

[6] J. Dai,S. Lin, D. Cheng, S. Zou, X. Shuai, "Interlayer-crosslinked micelle with partially hydrated core showing reduction and pH dual sensitivity for pinpointed intracellular drug release," *Angew. Chem. Int. Ed. Engl.,* vol. 50, pp. 9404-9408, 2011.

[7] J. Karvinen, T.O. Ihalainen, M.T. Calejo, I. Jönkkäri, M. Kellomäki, "Characterization of the microstructure of hydrazone crosslinked polysaccharide-based hydrogels through rheological and diffusion studies," *Materials Science and Engineering: C*, 2019. **94**: p. 1056-1066.

[8] P.B. Welzel, S. Prokoph, A. Zieris, M. Grimmer, S. Zschoche, U. Freudenberg, C. Werner, "Modulating biofunctional starPEG heparin hydrogels by varying size and ratio of the constituents," *Polymers*, 2011. **3**(1): p. 602-620.

[9] M. Rubinstein, R.H. Colby, *Oxford University Press: New York.* NY, USA, 2003. **23**: p. 259.

[10] R. Suriano, G. Griffini, M. Chiari, M. Levi, S. Turri, "Rheological and mechanical behavior of polyacrylamide hydrogels chemically crosslinked with allyl agarose for two-dimensional gel electrophoresis", *Journal of the mechanical behavior of biomedical materials*, 2014. **30**: p. 339-346.
